# Efficient endogenous protein labelling in *Dictyostelium* using CRISPR/Cas9 knock-in and split fluorescent proteins

**DOI:** 10.1371/journal.pone.0326577

**Published:** 2025-06-20

**Authors:** Kensuke Yamashita, Tetsuya Muramoto

**Affiliations:** Department of Biology, Faculty of Science, Toho University, Funabashi, Chiba, Japan; Hirosaki University Graduate School of Medicine, JAPAN

## Abstract

Fluorescent protein tagging is a powerful technique for visualising protein dynamics; however, full-length fluorescent protein knock-in can be inefficient at certain genomic loci, making it challenging to achieve stable and uniform expression. To address this issue, we used CRISPR/Cas9-mediated knock-in strategies with split fluorescent proteins in *Dictyostelium discoideum*. This approach enabled efficient integration of the short mNeonGreen2 (mNG2) fragment, mNG2_11_, particularly at functionally critical loci such as major histone *h2bv3*, where full-length tagging was unsuccessful. Our analysis revealed that inserting tandem repeats of mNG2_11_ at the *h2bv3* locus progressively impaired cell proliferation, indicating that functional disruption depends on insert size. These findings suggest that using short tags like mNG2_11_ minimises functional interference and facilitates knock-in at sensitive loci. We further optimised the fluorescence intensity by fine-tuning the expression of the long fragment, mNG2_1–10_, and introducing tandem repeats of mNG2_11_. This approach provides a reliable method for precise and stable endogenous protein labelling, facilitating live-cell imaging and functional studies in *D. discoideum*.

## Introduction

Protein tagging is a fundamental technique in biological analysis that enables protein purification, interaction studies, and subcellular localisation analysis via biochemical assays and microscopy. Common tagging strategies include fluorescent proteins, such as GFP, self-labelling enzymes, and short epitope tags composed of a small number of amino acid residues [1–3]. Typically, these tags are fused to target proteins and expressed in plasmid vectors under strong promoters. However, overproduction induced by these strong promoters can lead to unintended consequences, including protein misfolding, aberrant localisation, protein aggregation, and disruption of endogenous functions [4–7].

To overcome these limitations, knock-in techniques enable the direct integration of tagged sequences into the genome, facilitating endogenous protein labelling. *Saccharomyces cerevisiae* was one of first organisms to undergo genome-wide tagging of endogenous proteins through homologous recombination [8]. Recently, advances in CRISPR/Cas9 genome editing and improved fluorescent protein designs have enabled high-throughput knock-in strategies in human cell lines such as HEK293 and induced pluripotent stem cells [9,10]. A key innovation in this field is the development of self-complementing split fluorescent proteins, wherein a full-length fluorescent protein is divided into two fragments that reassemble within the cell to restore fluorescence. Several split systems have been established, including GFP_1–10_/GFP_11_, sfCherry_1–10_/sfCherry_11_, and mNeonGreen2_1–10_/mNeonGreen2_11_ (mNG2_1–10_/mNG2_11_) [11,12]. This approach enables the efficient labelling of endogenous proteins by incorporating small split-fragment sequences into the genome. The knock-in donor sequence is often synthesised as a short single-stranded oligodeoxynucleotide (ssODN) of <200 nucleotides (nt), which in some systems have been reported to improve knock-in efficiency [13,14]. However, the relationship between donor size and knock-in success may also be influenced by other factors such as sequence composition, chromatin accessibility, and the functional impact of inserted sequences.

Model organisms have been instrumental in advancing biological research, with *Dictyostelium discoideum* serving as a valuable system that bridges the biological complexity between yeast and complex eukaryotes. This organism is genetically tractable and shares numerous homologous genes with other species, making it an effective model for studying processes such as chemotaxis, DNA damage repair, autophagy, cell-autonomous immunity, and neurodegenerative diseases [15–18]. Previously, we established a highly efficient CRISPR/Cas9-mediated genome editing system for *D. discoideum*, including optimised methods for AT-rich genomic regions and whole-genome screening [19–22]. However, gene knock-in remains challenging owing to several factors, including the frequency of successful knock-in events, the complexity of donor DNA preparation, and the potential lethality associated with tagging essential genes.

To address the limitation in the frequency of successful integration events, we utilised split fluorescent proteins, specifically mNG2, to improve the knock-in efficiency in *D. discoideum*. To further enhance the expression stability, we explored the use of safe harbour loci, such as *cinD* and *scdB* [23], as genomic integration sites for the long fragment mNG2_1–10_. These loci allow for stable transgene expression while minimising disruption to endogenous gene function, thereby reducing variability in expression levels among cells. Based on these findings, we propose a novel knock-in strategy using split fluorescent proteins to generate labelled *D. discoideum* cell lines.

## Results

### Exploring molecular dynamics through fluorescent protein knock-in with minimal impact on cellular function

**Fig 1:**
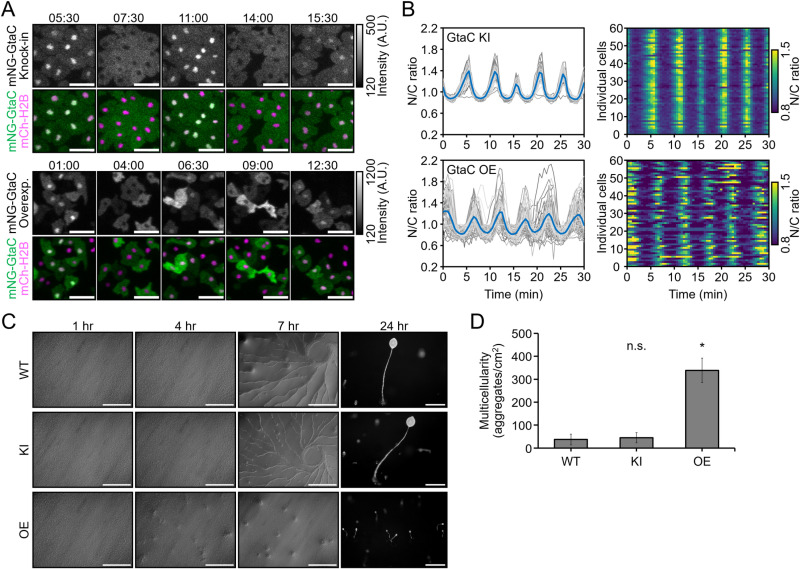
Analysis of mNG-GtaC fusion protein dynamics in knock-in and overexpressing cells. (A) Visualisation of mNG-GtaC dynamics during the aggregation stage. Images were taken every 30 s starting at 5 h after starvation. The upper panels show cells with mNG knock-in at the *gtaC* locus, while the lower panels depict cells overexpressing mNG-GtaC from a plasmid. Grayscale images represent fluorescence from mNG signals, and colour images display merged mNG and mCherry channels, using cells in which the nucleus was labelled with histone mCherry-H2Bv3. Nuclear mCherry-H2Bv3 expression was achieved by knock-in of the [*act15*]:mCherry-H2Bv3 cassette at the *cinD* locus. Fluorescence intensity in the grayscale images is indicated by the colour key. Time is in min:sec. Scale bars: 20 µm. (B) Nuclear-to-cytoplasmic (N/C) ratio of mNG-GtaC after 5 h of development. Data represent individual measurements from 60 cells, with the mean value indicated. (C) Differences in multicellular formation between knock-in (KI) and overexpressing (OE) cells. Scale bars: 0.5 mm. (D) Comparison of aggregate formation in knock-in and overexpressing cells at 7 h of development. *n* = 48, 38, 17 images. n.s., *p* > 0.05, *p < 0.001; The Kruskal–Wallis test followed by Steel’s post hoc test.

GtaC is a transcription factor that exhibits periodic nuclear-cytoplasmic shuttling in response to cAMP oscillations during early development. Its subcellular localisation was previously visualised using fluorescent protein tagging, where tagged GtaC was expressed under a strong constitutive promoter from a high-copy extrachromosomal vector [24]. To capture this oscillatory behaviour, time-lapse imaging was performed at 5 h after starvation, when cells initiate aggregation and cAMP oscillations emerge. In the newly generated mNeonGreen (mNG)-GtaC overexpression strain, fluorescence intensity varied between individual cells, likely because of the differences in the vector copy number (Fig 1A). Nevertheless, GtaC exhibited periodic nuclear-cytoplasmic shuttling, consistent with the previous report. In this study, we used the CRISPR/Cas9 system to tag endogenous GtaC with a fluorescent protein (S1 Fig), enabling direct comparison with the overexpression strain. Although single-copy *gtaC* expression resulted in weaker fluorescence than overexpression, GtaC localisation dynamics were clearly observable and more precisely defined, as the knock-in strain exhibited highly synchronised nuclear-cytoplasmic shuttling (Fig 1B). Furthermore, while knock-in cells exhibited normal post-starvation multicellular development, GtaC overexpressing cells underwent precocious development, forming smaller fruiting bodies and a significantly higher number of aggregates per unit area compared to those in wild-type (WT) and knock-in (KI) cells (Fig 1C, 1D). These findings indicate that fluorescent protein knock-in preserves the native dynamics of GtaC localisation and avoids overexpression-induced artefacts, such as asynchronous nuclear localisation and developmental abnormalities.

### Visualisation of endogenous proteins and cell labelling via CRISPR-mediated knock-in of full-length fluorescent tags

**Fig 2:**
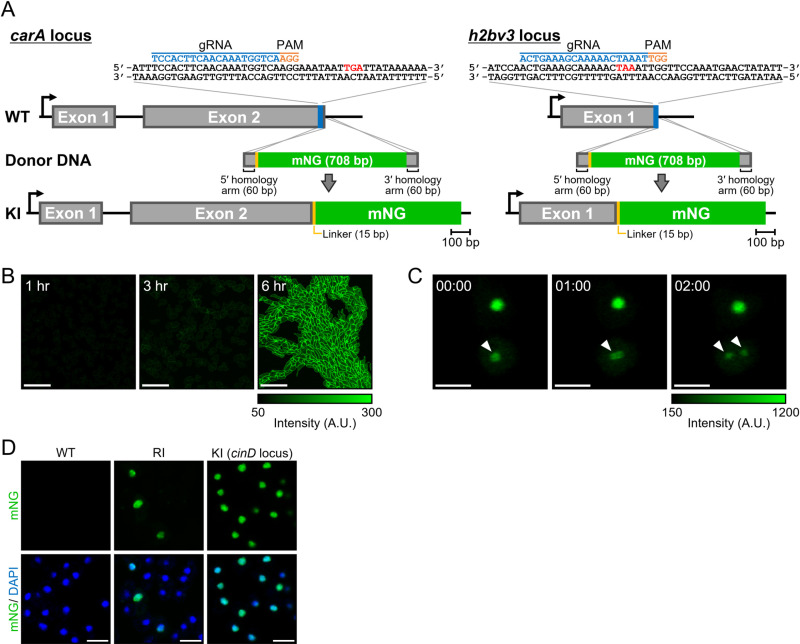
Knock-in of full-length fluorescent tags for visualisation of endogenous proteins. (A) Schematic representation of knock-in targeting at the *carA* and *h2bv3* loci using a full-length fluorescent tag. Expanded C-terminal sequences of the target genes are shown, with the gRNA indicated in blue, PAM sequence highlighted in orange, and stop codon marked in red. (B) Plasma membrane fluorescence of cAR1 during early developmental stages. Scale bars: 50 µm. (C) Nuclear labelling of H2Bv3 and monitoring of the mitotic process. Arrowheads indicate dividing cells, as evidenced by the dynamic redistribution of nuclear fluorescence. Time is in min:sec. Scale bars: 10 µm. (D) Representative images of mNG fluorescence in (wild-type [WT]; no fluorescence), a randomly integrated H2Bv3-labelled clone (RI), and mNG-fused H2Bv3 knocked in at the *cinD* locus (KI) under the control of a constitutive promoter. Upper panels show mNG fluorescence alone, whereas lower panels display merged mNG and DAPI signals. All nuclei within the field are visualised by DAPI staining. Scale bars: 10 µm.

To expand the application of CRISPR/Cas9-mediated knock-in of genes for endogenous protein visualisation, we targeted *carA*, which encodes a cAMP receptor localised to the plasma membrane, and *h2bv3*, which encodes a major histone H2B variant observed in the nucleus (Fig 2A). Knock-in of mNG at the *carA* locus was confirmed using PCR with an efficiency of 2.1% (2/92 clones). Here, knock-in efficiency is defined as the percentage of PCR-positive clones among all screened clones, reflecting both successful integration events and subsequent cell survival. Fluorescence at the plasma membrane became detectable approximately 3 h after starvation and increased by 6 h, demonstrating a uniform distribution across cells (Fig 2B). This temporal pattern is consistent with previous reports of *carA* expression, which is upregulated during early development and peaks around 6 h [25].

In contrast, no PCR-positive knock-in clones were obtained for *h2bv3* (0/128 clones). However, since a small fraction of cells (<0.5%) in the bulk-transfected population exhibited nuclear fluorescence, we hypothesised that rare knock-in-positive clones or cells with random integration could still be isolated based on fluorescence. We therefore screened over 300 individual clones based on fluorescence and identified a strain showing H2Bv3-like nuclear dynamics (Fig 2C) [26,27]. However, PCR analysis revealed that although the mNG sequence was present, it was not inserted at the *h2bv3* locus, raising the possibility of genomic rearrangement following initial integration. Furthermore, only ~12% of cells in the clone showed fluorescence, and this heterogeneity persisted after re-cloning (Fig 2D). In contrast, knock-in at safe harbour loci such as *cinD* and *scdB* under constitutive promoters yielded uniform nuclear fluorescence across cells (Fig 2D and S2 Fig). We attempted inverse PCR using primers targeting the mNG cassette to identify the integration site but were unsuccessful, likely due to complex tandem insertions into repetitive elements such as tRNA clusters, which are known hotspots for random integration in *Dictyostelium* [28–30]. These elements are known to cause expression variability through epigenetic silencing [31,32], potentially explaining the observed heterogeneity. Together, these findings from attempts at endogenous knock-in demonstrate that full-length knock-in at functionally critical loci such as *h2bv3* is technically and biologically challenging, reinforcing the need for alternative strategies such as split fluorescence tagging.

### Analysis of fluorescence intensity and localisation of split-mNeonGreen2 in *Dictyostelium*

**Fig 3:**
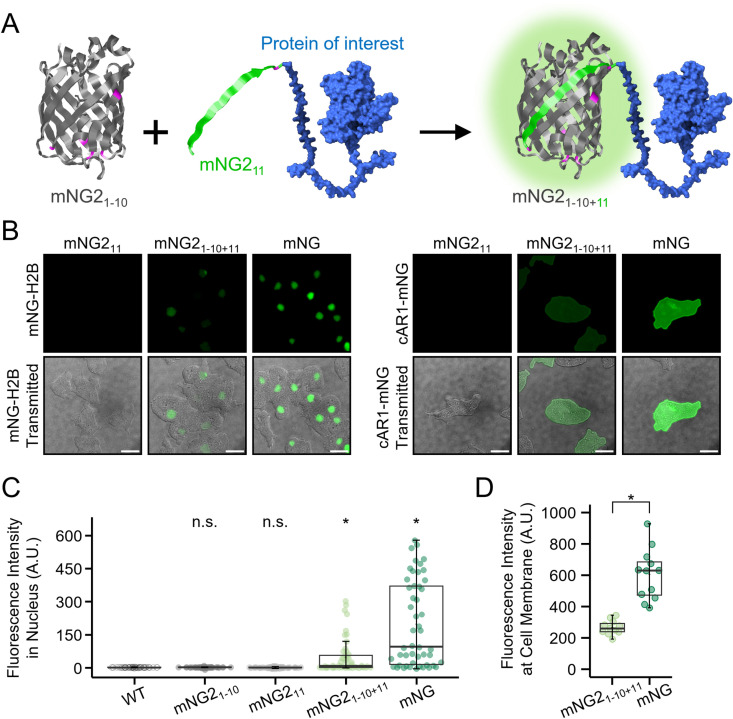
Analysis of fluorescence intensity and cellular localisation of split-mNG2. (A) Schematic representation of the split-mNG2 system, showing the separation of mNG2_1-10_ and mNG2_11_ fragments. (B) Comparison of split and full-length fluorescent proteins fused to H2Bv3 and cAR1. Cells expressing only the short fragment (mNG2_11_), both fragments (mNG2_1-10_ + mNG2_11_), or full-length mNG (mNG) were analysed. H2B-expressing cells were observed immediately after starvation condition, whereas cAR1-expressing cells were observed at 7 h of development. Scale bars: 10 µm. (C) Comparison of fluorescence intensity between split-mNG2 and full-length mNG. Nuclei were stained with DAPI, and green fluorescence signals were measured within the DAPI-defined nuclear region. Fluorescence intensities were measured after background subtraction using the average signal from a cell-free area. *n* = 50, n.s., *p* > 0.05, *p < 0.001; The Kruskal–Wallis test followed by Steel’s post hoc test. (D) Quantification of plasma membrane fluorescence intensity in live cAR1-mNG-expressing cells. Mean fluorescence intensity was measured and compared between the split-mNG2 and full-length mNG. *n* = 11 for mNG2_1-10+11_; 12 for mNG. *p < 0.001; Welch’s t-test.

To assess the functionality of split fluorescent proteins in *Dictyostelium*, we first examined their performance using an overexpression system. Although the split-tag strategy improves the feasibility of knock-in procedures [14], it may result in reduced fluorescence intensity and potential aggregation if the two fragments misassemble in *Dictyostelium* cells. To systematically evaluate fluorescence efficiency and localisation fidelity, we constructed expression vectors encoding split mNG2 fragments. The short fragment, mNG2_11_ (16 amino acids), was fused to nuclear-localised H2Bv3 or plasma membrane-localised cAMP receptor cAR1, whereas the long fragment, mNG2_1–10_, was expressed separately from a different vector (Fig 3A). Cells expressing only one of the two fragments showed no detectable fluorescence, confirming that both fragments are required for signal generation. In contrast, co-expression of mNG2_11_-H2Bv3 and mNG2_1–10_ resulted in clear nuclear fluorescence (Fig 3B), with the signal intensity reaching approximately 50% of that observed in cells expressing full-length mNG-H2Bv3 (Fig 3C). A similar result was observed for cAR1; as the fluorescence from cAR1-mNG2_11_ and mNG2_1–10_, although weaker than that of full-length cAR1-mNG (Fig 3D), remained reliably detectable and correctly localised at the plasma membrane (Fig 3B). To justify the use of full-length mNG as a reference, we compared its fluorescence to that of full-length mNG2 in *Dictyostelium*. Although both are derived from the same fluorescent protein sequence, our overexpression analysis demonstrated that mNG2 exhibits slightly lower fluorescence than mNG (S3 Fig), consistent with previous findings [12]. Therefore, we selected full-length mNG as a practical reference for evaluating the fluorescence output of split-mNG2 constructs.

### CRISPR/Cas9-mediated knock-in of split-mNG2 into endogenous gene loci

**Fig 4:**
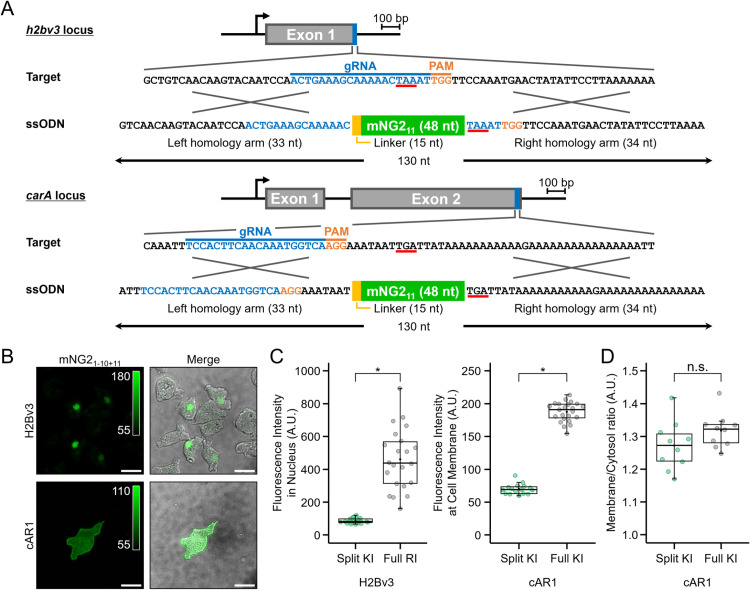
Visualisation of endogenous proteins through the knock-in of split mNG2_11_. (A) C-terminal sequences of the knock-in target loci and ssODNs used as knock-in donors. The genomic sequences of *h2bv3* and *carA* are shown, with the gRNA indicated in blue, PAM sequence highlighted in orange, and the stop codon underlined in red. The knock-in donor consists of a 130-nt ssODN, containing a homologous left arm, right arm, linker, and mNG2_11_. The short fragment, mNG2_11_, was knocked into the target gene, whereas the long fragment, mNG2_1-10_, was stably expressed from a plasmid under control of the *act15* promoter. (B) Fluorescence images of knock-in clones. mNG2_1-10_ was overexpressed from an expression vector. Cells expressing H2Bv3-mNG2 were imaged immediately after starvation, whereas cells expressing cAR1-mNG2 were imaged at 7 h of development. Scale bars: 10 µm. (C) Comparison of fluorescence intensity between split mNG2 and full-length mNG. Full-length mNG was tagged at the C-terminus of H2Bv3 (random integration) and cAR1 (knock-in) and used as a control for fluorescence intensity comparison. H2Bv3 (*n* = 21 cells for split, *n* = 23 cells for full-length), cAR1 (*n* = 16 cells for split, *n* = 23 cells for full-length). *p < 0.001; Wilcoxon rank-sum test. (D) Comparison of membrane-to-cytoplasm fluorescence intensity ratios of cAR1 between split mNG2 and full-length mNG (*n* = 10 cells). n.s., *p* > 0.05; Wilcoxon rank-sum test.

To visualise endogenous proteins using split fluorescent tags, we knocked in mNG2_11_ at the *carA* and *h2bv3* loci using CRISPR/Cas9. The donor DNA, synthesised as a 130-nt ssODN, included a five-amino-acid glycine-serine linker between the left homology arm and mNG2_11_ (Fig 4A). The knock-in efficiencies determined by PCR were 32.3% (31/96 clones) for *h2bv3* and 1.3% (3/232 clones) for *carA* (S4A, S4B Fig). Although knock-in efficiency for *carA* was relatively low, successful integration was achieved, even with the right homology arm being 94% AT-rich and only 34-nt long (Fig 4A). To investigate how donor design, such as homology arm length and molecular structure (ssODN vs dsDNA), affects knock-in efficiency, we amplified donor DNAs by PCR with varying homology arm lengths. The resulting knock-in efficiencies were 0.78% (1/128 clones) for 33- and 34-bp arms, 10.94% (14/128 clones) for 48- and 49-bp arms, and 1.56% (2/128 clones) for 68- and 69-bp arms (S4C Fig). Although some improvement was observed with intermediate arm lengths, no clear trend was seen across all constructs. These results suggest that donor design does not fully determine knock-in efficiency and that locus-specific factors may play a more significant role. These results indicate that both donor configuration and the genomic context influence knock-in success, and optimisation strategies may need to be tailored accordingly. Notably, although full-length knock-in at *h2bv3* was unsuccessful, using the split fluorescent protein system enabled efficient knock-in at this locus, highlighting its utility for tagging difficult-to-edit genes. Cells with mNG_11_ knock-in at *carA* and *h2bv3* exhibited appropriate plasma membrane and nuclear localisation, respectively, without detectable unintended protein aggregation (Fig 4B). Although the fluorescence intensity of the split-mNG2 constructs was lower than that of the full-length mNG-tagged protein, it remained sufficient for analysing protein dynamics (Fig 4C). To further assess the fidelity of membrane localisation in split-tagged cAR1, we quantified the membrane-to-cytoplasm fluorescence intensity ratio, which revealed comparable spatial distribution between split and full-length tagging strategies (Fig 4D).

### Tandem repeats of split mNG2 fragments enhance fluorescence intensity

Tandem repeats of split fluorescent protein fragments are known to enhance fluorescence intensity by increasing reconstitution frequency and resistance to photobleaching [11]. To assess this in *Dictyostelium*, we constructed donor DNAs containing one, two, and three tandem repeats and integrated them into the *carA* and *h2bv3* loci. Because these constructs exceeded the length suitable for chemical synthesis, they were prepared as PCR-amplified dsDNA fragments, in contrast to earlier experiments that used short ssODNs. To avoid unintended recombination among tandem repeats within the donor DNA, repeat sequences were designed using synonymous codons that preserve the same amino acids but reduce nucleotide-level homology (S5A Fig). PCR analysis confirmed a knock-in efficiency of approximately 1.5% at the *carA* locus, with no significant differences between repeat constructs (S5B Fig). However, knock-in efficiency at the *h2bv3* locus declined to 2.4% (7/288 clones) and 0.7% (4/576 clones) for constructs containing two and three repeats, respectively. Notably, although several clones were PCR-positive for the three-repeat construct, nucleotide sequencing revealed that none contained the complete intended insertion.

**Fig 5:**
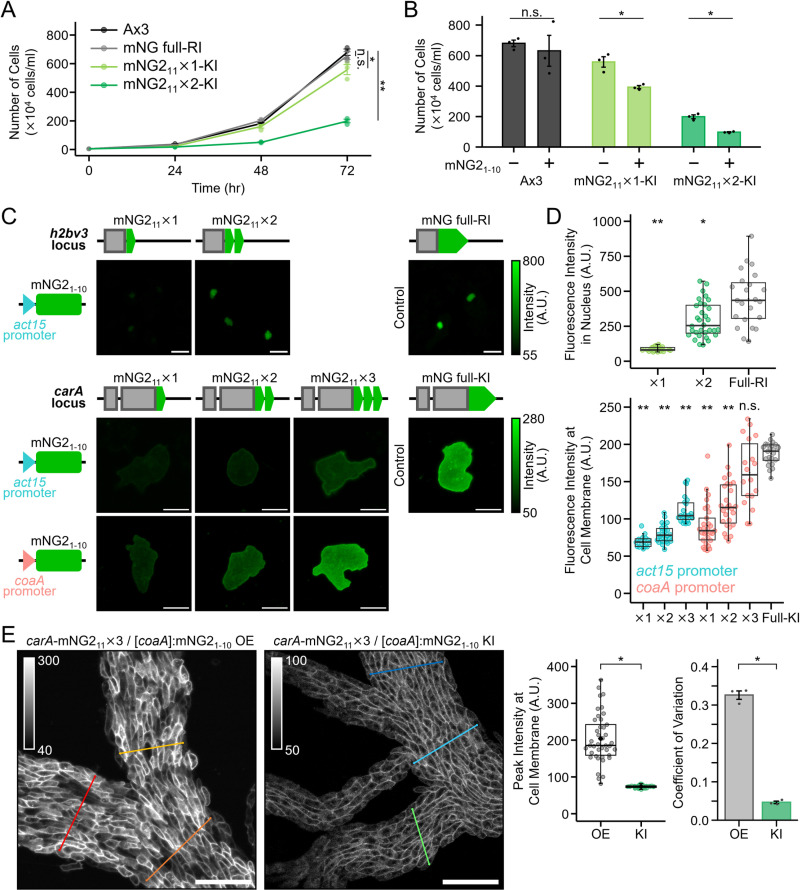
Enhancement of fluorescence intensity and homogeneity by tandem repeats of split mNG2_11_. (A) Growth curves of cells with one or two tandem repeats of mNG_11_ knocked into the *h2bv3* locus. Data points represent the mean ± SE of three biological replicates, with individual data points shown. n.s., *p* > 0.05, *p < 0.05, ***p < 0.001; The Kruskal–Wallis test followed by Dunnet post hoc test between final samples at 72 hours. (B) Effect of mNG2 reconstitution by mNG2_1-10_ expression on the proliferation of *h2bv3* knock-in cells with tandem mNG2_11_ repeats. Proliferation at 72 h was compared. n.s., *p* > 0.05, *p < 0.01; Student’s t-test. (C) Fluorescence images of cells with different numbers of mNG2_11_ repeats knocked into the *h2bv3* and *carA* loci. mNG2_1-10_ was stably expressed from a plasmid under either the *act15* or *coaA* promoter. Images are displayed with brightness corresponding to the colour key. Cells expressing endogenous H2Bv3 or cAR1 were visualised at 0 or 7 h of development, respectively. Scale bars: 10 µm. (D) Comparison of fluorescence intensities across different tandem repeat constructs. The top panel shows H2Bv3 constructs including the full-length mNG expressed from a randomly integrated (RI) locus, whereas the bottom panel shows *carA* knock-in (KI) constructs (*n* ≥ 16 cells per condition). The full-length mNG-tagging was used as a practical reference to evaluate the achievable fluorescence intensity of mNG2-based split tags. n.s., *p* > 0.05, *p < 0.01, **p < 0.001; The Kruskal–Wallis test followed by Steel’s post hoc test. (E) Knock-in of [*coaA*]:mNG2_1-10_ at the *cinD* locus improves the heterogeneity of cAR1 fluorescence at the plasma membrane during aggregation. The left graph shows the peak fluorescence intensities measured along the cell membrane, and the right graph displays the coefficient of variation (CV) calculated from these measurements. Scale bars: 50 µm. *p < 0.001; Welch’s t-test.

To investigate whether these integration events affected cell physiology, we next analysed cell proliferation. In the absence of mNG2_1–10_ expression, insertion of mNG2_11_ at the *h2bv3* locus impaired growth, with the two-repeat construct exhibiting a more pronounced growth delay than the one-repeat construct (Fig 5A). When mNG2_1–10_ was co-expressed, a moderate reduction in growth was also observed, suggesting that reconstitution of the fluorescent protein may further impair H2Bv3 function and reduce viability (Fig 5B). Together, these findings indicate that knock-in success at sensitive loci such as *h2bv3* is primarily influenced by insert size, while the extent of fluorescent protein reconstitution may also contribute to reduced cellular viability.

The fluorescence intensity increased with the repeat number for both nuclear-localised H2Bv3 and membrane-localised cAR1 under plasmid-based expression of mNG2_1–10_ (Fig 5C). Notably, the insertion of two repeats at *h2bv3* locus increased the fluorescence by 3.5-fold, whereas insertion of three repeats at *carA* enhanced fluorescence by 2-fold compared to a single repeat. However, the fluorescence intensity from *carA* knock-in strain with three tandem repeats remained at approximately 50% of that observed with full-length mNG knock-in cells (Fig 5D). To further enhance fluorescence, we replaced the *act15* promoter driving mNG2_1–10_ expression with the stronger *coaA* promoter [25], which improved expression levels and increased the reconstitution efficiency of the split fragments. Although this modification led to higher fluorescence, it also introduced cell-to-cell variability (Fig 5D). To mitigate this variability, we integrated the [*coaA*]:mNG2_1–10_ at the *cinD* locus provided stable expression (S6 Fig). To evaluate the applicability of this strategy in a physiologically relevant context, we examined fluorescence at the plasma membrane in multicellular aggregates, where cAR1 plays a critical role during development. We quantified the intensity of membrane fluorescence in aggregated cells, confirming that stable expression from the *cinD* locus maintained sufficient brightness for developmental imaging (Fig 5E). Furthermore, the distribution of fluorescence intensity along the plasma membrane appeared more homogeneous in knock-in cells compared to those with plasmid-based overexpression. This observation was supported by coefficient of variation (CV) analysis, which revealed reduced fluorescence variability in the knock-in strains (Fig 5E). Together, these results demonstrate that combining tandem repeat knock-in with optimised expression of mNG2_1–10_, achieved through careful promoter selection and genomic integration, enables robust and uniform fluorescence suitable for functional live imaging during *Dictyostelium* development.

## Discussion

Overexpression of key regulatory proteins, such as GtaC, can disrupt molecular networks, leading to unpredictable effects and complicating the precise determination of their specific functions. Fluorescent tag knock-in at endogenous gene loci provides a more reliable alternative by preserving natural expression patterns and avoiding artefacts associated with overexpression. However, full-length fluorescent protein knock-in can be inefficient at certain genomic loci, limiting its applicability. In this study, attempts to knock in a full-length fluorescent tag at the *h2bv3* locus, which encodes a major nucleosomal H2B in *Dictyostelium* [33,34], were unsuccessful. This likely reflects strong functional interference caused by full-length tagging of H2Bv3, which plays an essential role in chromatin stability and cellular viability. In mammalian systems, similar challenges have been reported. For example, full-length fluorescent tagging of the *HIST2H2BE* locus, one of several redundant H2B genes, resulted in a low knock-in efficiency of 1.6% [35]. Unlike human cells, which express multiple H2B variants that may compensate for gene disruption, *Dictyostelium* relies predominantly on H2Bv3 [33,34]. Thus, functional disruption of this essential histone is more likely to impair cell viability, highlighting how both gene essentiality and genomic context can influence knock-in outcomes. However, by employing split fluorescent proteins, specifically mNG2, we successfully incorporated the short fragment mNG2_11_ at endogenous loci, thereby significantly improving knock-in. Additionally, the use of chemically synthesised short ssODN donor fragments (< 200 nt) simplifies the experimental workflow by eliminating the need for complex donor vector construction and purification. These findings demonstrate that even when full-length tagging is inefficient, shorter fluorescent tags remain a viable alternative for endogenous protein labelling.

Although split fluorescent proteins improve knock-in efficiency, their fluorescence intensity is often lower than that of full-length tags. This is not due to reduced intrinsic brightness, as split mNG2 retains single-molecule brightness comparable to full-length mNG2 [12,47]. Rather, the overall signal is lower likely because reconstitution is incomplete in cells. To address this limitation, we optimised the promoter driving mNG2_1–10_ expression and introduced tandem repeats of mNG2_11_, which substantially increased the signal intensity. With these optimisations, introducing three tandem repeats of mNG2_11_ alongside [*coaA*]:mNG2_1–10_ at a safe harbour locus resulted in sufficient fluorescence intensity for reliable imaging analysis. This strategy enables clear visualisation of tagged proteins without the need for full-length fluorescent protein expression. Notably, integration into safe harbour loci, such as *cinD*, *scdB*, and *act5* [23,36,37], resulted in uniform expression across cells, reducing cell-to-cell variability. In contrast, overexpression of mNG2_1–10_ from a plasmid remains the most practical option for obtaining bright fluorescence signals; however, this approach introduces variability among cells owing to differences in the plasmid copy number and expression levels.

Further improvements in fluorescence intensity can be achieved by refining mNG2_1–10_ variants or exploring alternative engineered fluorescent proteins with higher brightness and stability. Tandem repeat strategies remain promising [11,13,38,39], although increasing repeat numbers may reduce knock-in efficiency because of the larger size of insert. Moreover, a higher number of repeats may disrupt the function of the target protein, possibly because of both the insert size and enhanced reconstitution efficiency of the split fluorescence protein. At the *h2bv3* locus, two-repeat knock-ins showed slower cell proliferation, suggesting that both larger insert size and functional interference contributed to lower knock-in success. These findings highlight the importance of balancing tag design and cellular viability, particularly when targeting essential genes. Alternatively, using shorter full-length fluorescent proteins may help mitigate these issues. For example, mStayGold and miRFP670nano3, which is smaller than mNeonGreen [40,41], could be a promising candidate for full-length knock-in, particularly at sensitive genomic loci. Future studies comparing fluorescent proteins of varying lengths and sequence characteristics will be valuable for further optimising knock-in strategies across different contexts.

The growing demand for multicolour live-cell imaging has led to advancements in fluorescent protein colour variations and microscopy techniques [2,42–46]. Nuclear labelling is essential for single-cell tracking during live-cell imaging, particularly in highly motile organisms like *Dictyostelium* [27,36]. The use of multiple fluorescent protein variants enables multicolour imaging and facilitates the distinction of subpopulations. Importantly, by targeting safe harbour loci, such nuclear labels can be stably introduced without disrupting endogenous gene function, providing a robust and flexible platform for multicolour imaging and reliable cell tracking during developmental processes. Although we employed mNG2 for labelling, other split fluorescent proteins, such as split-sfCherry (red) and split-Cerulean (cyan), have been developed and applied in mammalian systems [47,48]. Implementing these alternative colour variants in *Dictyostelium* may enable true multicolour imaging of multiple endogenous proteins within the same cell, facilitating more complex dynamic analyses. Additionally, tandem repeats of split fluorescent tags can optimise fluorescence channel usage, allowing cells to be distinguished based on differences in brightness. By leveraging fluorescence intensity variation among one, two, and three tandem repeats, even cells expressing the same fluorescent protein colour can be distinguished. This strategy expands the potential of single-colour imaging and enables multi-population tracking within the same fluorescence channel. Furthermore, it allows for the identification of subpopulations within seemingly homogeneous cell populations as visualised under microscope, providing a means to investigate how cellular heterogeneity influences fate decisions. Applying this method may reveal how factors such as nutrient history and cell cycle position contribute to developmental trajectories in *Dictyostelium* [49,50], offering deeper insights into cell fate bifurcation.

Split fluorescent tags are particularly valuable for detecting organelle contacts and cell-cell adhesion events mediated by membrane protein interactions, which are often challenging to study using biochemical methods [51–53]. Additionally, split fluorescent proteins enable the analysis of membrane protein interactions in living cells, allowing for real-time monitoring of dynamic protein-protein associations in their native cellular contexts. Unlike conventional biochemical approaches, which often require cell lysis and immunoprecipitation, use of split fluorescence tags preserves the cellular environment while capturing interaction dynamics. In *Dictyostelium*, the application of split fluorescent tags is expected to improve studies of chemotactic signalling mediated by membrane receptors and multicellular organisation driven by cell adhesion molecules.

This study demonstrates that fluorescent protein knock-in enables stable and precise endogenous protein labelling while minimising phenotypic artefacts and cytotoxicity associated with overexpression under strong promoters. Furthermore, when full-length knock-in presents challenges, split fluorescent tags provide an efficient alternative for achieving successful knock-in. With further optimisation and integration with advanced imaging techniques, split fluorescent proteins will continue to serve as a powerful tool for functional studies in *Dictyostelium* and other model organisms.

## Materials and methods

### Generation of expression vectors

The green fluorescent protein mNG fragment was amplified using primers containing BglII and BamHI restriction sites at each end and ligated into the BglII sites of the extrachromosomal vectors pDM358 and pDM304, generating pTM2045 and pTM2046. To construct pTM2066 and pTM2609, the BglII/SpeI sites at the C-terminus of mNG in these vectors were digested, and the *gtaC* and *h2bv3* genes were fused into these sites. Similarly, pDM1208 was digested with BglII/SpeI, and H2B was inserted at the C-terminus of mCherry to generate pTM1931. For *carA,* tagging with a C-terminal linker was necessary to ensure proper folding and functional expression. Therefore, pTM2638 was generated by fusing a five-amino acid linker (GGSGG)-mNG fragment with a SpeI/XbaI site into SpeI-treated pDM304. The plasmid pTM2659 was generated by inserting the *carA* gene into the BglII/SpeI site of pTM2638. Optimised versions of mNG2 and mTagBFP2 (mTB2) for *Dictyostelium* codon usage (S1 Table), were generated by a gene synthesis service (IDT) and cloned using In-Fusion (TaKaRa Bio.) into the cloning vector pBlueScript II SK(+). The constructs were subsequently cloned into pDM304 or pDM326, generating expression vectors pTM2054 and pTM2119. The mNG2_1−10_ fragment was PCR-amplified using mNG2 as a template and the resulting PCR product was cloned into pDM304 or pDM358 via the BglII/SpeI sites, generating pTM2055 or pTM2672. While pTM2055 was primarily used for split fluorescent protein experiments, pTM2672 was specifically used for split-tagging of cAR1 in Fig 4. For split-mNG constructs, mNG2_11_ was fused to the N-terminus of *h2bv3* or C-terminus of *carA* by adding the mNG2_11_ sequence to the ends of the PCR primers, and the resulting products were introduced into pDM358 to generate pTM2652 and pTM2653. A tandem repeat of cAR1-mNG2_11_ × 2 (pTM2690) was generated by inverse PCR using pTM2653 as a template. Similarly, pTM2691, which contains cAR1-mNG2_11_ × 3, was constructed using pTM2690 as a template. Knock-in donors for H2Bv3-mNG2_11_ × 2 and ×3 were PCR-amplified using pTM2690 and pTM2691 as templates and inserted into pMD20 (TaKaRa Bio.) by TA cloning (pTM2783 and pTM2784). HistoneH1 or mNG2_1−10_ was inserted into the N-terminus of mTB2 via the GGSGG linker or self-cleaving peptide P2A (pTM2580 and pTM2701). To increase the fluorescence intensity of mTB2, a (GGSGG)-mTB2 fragment with a SpeI/XbaI site was fused to SpeI-treated pTM2580 to create pTM2585 in tandem with two mTB2 molecules. Finally, the H1-mTB2 × 2 fragment digested at the BglII/HindIII site from pTM2585 was fused to the same site of pTM2554 for expression downstream of the *coaA* promoter (pTM2595). The expression vectors and primers used for plasmid cloning are listed in S2 Tables and S3.

### Generation of knock-in mutants using CRISPR/Cas9 techniques

Knock-in cell lines were generated using CRISPR/Cas9 with gRNAs specifically designed for the *gtaC*, *cinD*, *scdB, carA*, and *h2bv3* loci [54]. The gRNAs were chemically synthesised as paired oligonucleotides containing AGCA or AAAC overhangs (S4 Table) and inserted into all-in-one CRISPR/Cas9 vectors using the Golden Gate Assembly system (S5 Table) [55]. Donor DNAs for knock-in mutant generation were amplified via PCR using the primers and templates specified in S6 Tables and S7. The 130-nt ssODNs used for the knock-in of split mNG2_11_ were synthesised by Macrogen Inc. Each donor DNA fragment contained homologous sequences of ≥ 33-nt. For GtaC imaging, the cells were tagged with the red fluorescent protein mCherry for nuclear labelling.

### Cell culture, transformation, and identification of transformants

*Dictyostelium* AX3 cells were cultured at 22°C in HL5 axenic medium supplemented with streptomycin. To obtain transiently expressing Cas9 and sgRNAs cells, 10 µg of an all-in-one CRISPR vector was transformed into the cells using H50 buffer. The donor DNA used for the knock-in consisted of either 2.4 µg of dsDNA amplified by PCR or 4 µL of 10 µM ssODN. Prior to transformation, ssODNs were denatured at 95°C for 5 min and maintained at 4°C in a single-stranded form until use. Electroporation was performed as previously described [55]. Following electroporation, cells were cultured in HL5 for 7–24 h and maintained for another 1–2 days in HL5 containing 10 µg/mL G418. As the cells became rounded, they recovered in HL5 without G418 for recovery. Single clones were isolated by plating on SM agar plates and incubating for 4–6 days until plaques formed. Cells with a full-length mNG knock-in at the *h2bv3* locus were isolated as single clones by limiting dilution in a 96-well microplate. Genomic DNA from the single clones was isolated using lysis buffer consisting of 1 × Ex Taq PCR buffer (TaKaRa), 0.5% NP40 and 50 µg/mL Proteinase K, and the suspension was incubated at 56°C for 45 min, followed by 95°C for 10 min. The cell lysate served as a template for PCR screening, and subsequent sequencing analysis of the purified PCR products was performed to determine whether the knock-in was successful. The primers used for PCR screening and Sanger sequencing are listed in S4 Table. After thawing, cell stocks were maintained in the log phase to prevent transition to the stationary phase and were not cultured for ≥ 2 weeks to minimise variations in growth conditions. Thirty-two new cell lines were established following previously described methodology (S8 Table).

### Observation and counting of multicellular structures

Cells in the exponential growth phase (1.0–1.5 × 10^6^ cells/mL) were washed with KK2 phosphate buffer (16.5 mM KH_2_PO_4_, 3.9 mM K_2_HPO_4_) to induce starvation and initiate development. This time point was designated as 0-h of development. Subsequently, the cells were plated on 1.5% agar (1.5% w/v Difco Bacto-agar in KK2) in 35 mm plastic dishes at a density of 2.6 × 10^5^ cells/cm^2^ for developmental observation and 5.2 × 10^5^ cells/cm^2^ for aggregate counting. After 10 min of incubation, KK2 in the supernatant was carefully removed, and the cells were incubated at 22°C until a specific development time was reached. An inverted microscope (CKX41, Olympus) equipped with a CPlan 10 × /0.25 NA RC1 objective lens was used for observation during the first 7 h of development, whereas a stereo microscope (Leica S9D, Leica) was employed at 24 h of development when three-dimensional fruiting bodies were formed. To analyse multicellularity, the number of aggregates per cm^2^ was counted at the 7 h of development.

### Cell preparation and imaging

Development was initiated by plating cells on 1.5% agar at a density of 2.6 × 10^5^ cells/cm^2^, following a previously described method [54]. The cells were incubated in the dark at 22°C until the designated developmental time was achieved. Approximately 30 min before imaging, the agar was cut into approximately 1-cm squares. Subsequently, the cell-adhering surface of the agar was placed face-down in a glass-bottomed dish. Mineral oil was applied to the surface of the agar to prevent desiccation. To visualise the nuclei of cells labelled with the fluorescent tag H2Bv3, the cells were fixed in methanol for 10 min, and the nuclei were subsequently stained with DAPI.

Cells were imaged using an inverted confocal microscope system (Nikon A1R) equipped with both resonant and galvanometric scanners. For live-cell time-lapse imaging, a resonant scanner and piezo Z-drive were used. Specifically, GtaC dynamics were recorded at 30-sec intervals for 30 min at 5 h after starvation using a Z-stack of seven sections at 2-μm intervals. Imaging of H2Bv3-mNG (full RI) was performed in proliferating cells at 1-min intervals using the same Z-stack settings. For snapshot imaging of split knock-in constructs, a galvanometric scanner was used with three Z-sections at 1-µm intervals. During the imaging process, an oil immersion objective with Plan Fluor 40 × /1.30 NA and Plan Apo λ 60 × /1.40 NA (Nikon) were used. Fluorescent tags were visualised using the following excitation wavelengths: mTB2 with a 405-nm solid-state laser; mNG and mNG2 with a 488-nm laser; mCherry with a 561-nm laser; and miRFP670 with a 640-nm laser. DAPI-stained nuclear DNA was visualised using a 405 nm laser. For miRFP670-H2Bv3 visualisation, agar was supplemented with 50 µg/mL biliverdin (30891, Sigma-Aldrich). Approximately 30-min before imaging, 1-cm agar squares containing cells were placed face-down in glass-bottom dishes (Delta T Culture Dishes; Bioptechs) to allow imaging.

### Image analysis

The imaging data were analysed using Volocity software to quantify the fluorescence intensity at the cellular level. Nuclear markers were used to track GtaC shuttling, enabling automated cell tracking and N/C ratio quantification in individual cells. The brightness of H2Bv3-tagged mNG and mNG2_11_ was assessed relative to DAPI nuclear signal. For fluorescence quantification, background subtraction was applied using the mean intensity from a cell-free area within the same imaging field. To measure the fluorescent intensity of cAR1-fused tags localised at the cell membrane, fluorescent images were first segmented to identify individual cells as distinct objects using Volocity software. Each object was then eroded five times using the erosion function to define the cytoplasmic region. This cytoplasmic mask was subtracted from the original cell mask using the subtraction function, yielding a membrane-specific region. The average fluorescence intensity within this membrane region was then calculated. Line profiles were generated using a straight-line tool to quantify variations in fluorescence intensity across the plasma membrane. The coefficient of variation was calculated from the maximum fluorescence signal along the line to assess signal variability.

## Supporting information

S1 Fig Knock-in of a gene encoding mNG tag at the *gtaC* locus. (A) Schematic representation of the knock-in strategy for integrating mNG at the *gtaC* locus using the CRISPR/Cas9 system. (B) PCR validation of the knock-in strain using primers targeting the regions upstream and downstream of the mNG insertion site. The knock-in strain produced a PCR product with an increased length corresponding to the inserted mNG sequence. (C) Nucleotide sequencing of the *gtaC* locus in knock-in mutants confirmed successful integration, with the sequence encoding mNG highlighted in green.
(TIF)

S2 Fig Insertion of knock-in cassettes encoding nuclear labelling marker into genomic safe harbour loci. (A) gRNA design for the CRISPR/Cas9 system targeting genomically safe harbour loci. The genomic sequences of *cinD* and *scdB* are shown, with the start codon highlighted in red, gRNA sequence in blue, and PAM sequence in orange. Homology arms for the knock-in donor design near the target sites are also displayed. (B) PCR validation of the knock-in strain using primers targeting the regions upstream and downstream of the insertion site. The knock-in strain produced a PCR product with an increased length, corresponding to the size of the inserted knock-in cassette. This knock-in cassette included a histone gene (*h1* or *h2bv3*) fused to one of four fluorescent tags. The expression of these fusion genes is regulated by either the *coaA* or *act15* promoter and is terminated by the *act8* terminator. (C) Representative images of nuclear labelling during early development (2 h) and stream formation (6 h). Targeted integration at the safe harbour locus resulted in stable and uniform nuclear labelling without inducing developmental abnormalities. Scale bars: 40 µm.
(TIF)

S3 Fig Comparison of fluorescence intensity between overexpressed full-length of mNG and mNG2. (A) Snapshots of cells overexpressing full-length mNG and mNG2 from plasmids. The images show only the mNG or mNG2. Fluorescence intensity of mNG is indicated by the colour key. Scale bars: 50 µm. (B) Quantification of fluorescence intensity of overexpressed full-length mNG and mNG2. *n* = 1117 for mNG; 978 for mNG2. *p < 0.001; The Wilcoxon rank-sum test.
(TIF)

S4 Fig PCR screening and sequence analysis of split mNG2_11_ knock-in clones. (A) Detection of knock-in events using PCR amplification. Forward primers were designed for the C-terminal region of the target gene and reverse primers within mNG2_11_ were used. Knock-in positive clones were identified based on the presence of PCR-amplified products. (B) Sequence analysis of PCR-positive clones. The stop codon is underlined in red. Linker sequences are highlighted in yellow, mNG2_11_ green; PAM sequences orange; and mutations red. Correct clones are marked in bold, with the number of base errors indicated in parentheses next to the clone number. (C) Comparison of knock-in efficiency based on differences in homologous arm lengths. Donor DNAs were prepared as PCR-amplified double-stranded DNA (dsDNA) fragments with total lengths of 130, 160, or 200 nucleotides.
(TIF)

S5 Fig Design and PCR validation of tandem repeat insertion of split mNG2_11_ into *carA.* (A) Schematic representation of CRISPR/Cas9-mediated insertion of split mNG2_11_ at the C-terminus of *carA*. Donor DNAs with varying repeat numbers are shown. (B) PCR-based detection of knock-in events using primers targeting *carA* and a flanking region outside the gene (red arrows). Expected PCR product sizes: 1,127 bp for WT, 1,190 bp for one repeat, 1,253 bp for two repeats, and 1,316 bp for three repeats.
(TIF)

S6 Fig Stable expression of mNG2_1–10_ using the *coaA* promoter. The plasmid vector pTM2701 was constructed to express mNG2_1–10_ under the control of the *coaA* promoter. Donor DNA generated using this vector as a template was integrated into the *cinD* locus, enabling stable genomic expression.
(TIF)

S1 Table Codon-optimised nucleotide sequences for mNeonGreen2 and mTagBFP2. (PDF)

S2 Table Expression vectors used in this study. mNG: mNeonGreen; mTB2: mTagBFP2; Blast: Blasticidin S; Hyg: Hygromycin.
(PDF)

S3 Table Oligonucleotides for cloning primers. Lowercase letters are any three nucleotides or restriction enzyme recognition sites. mNG: mNeonGreen; mTB2: mTagBFP2.
(PDF)

S4 Table Oligonucleotides for gRNAs and sequencing primers. Lowercase letters of the gRNA represent overhang sequences for Golden Gate Assembly. mNG: mNeonGreen.
(PDF)

S5 Table CRISPR/Cas9 vectors for generating knock-in mutants. (PDF)

S6 Table Primers for generating donor DNAs. Lowercase letters represent homologous sequences of target genes. mNG: mNeonGreen.
(PDF)

S7 Table Plasmid DNAs used as PCR templates. mNG: mNeonGreen; mTB2: mTagBFP2.
(PDF)

S8 Table Cell lines used in this study. KI: Knock-in; OE: Overexpression; Hyg: Hygromycin B. mNG: mNeonGreen; mTB2: mTagBFP2.
(PDF)

S1 Raw images Raw gel electrophoresis images corresponding to the Supplementary Figures in the paper. (A) The original gel for S1B Fig. (B) The original gel for Fig S2B. (C) The original gel for Fig S4A. (D) The original gel for S5B.
(PDF)
