# Supplementary figures and images for: Efficient endogenous protein labelling in Dictyostelium using CRISPR/Cas9 knock-in and split fluorescent proteins
Source: PLoS One. 2025 Jun 20;20(6):e0326577. doi: 10.1371/journal.pone.0326577 (PMC12180633; doi:10.1371/journal.pone.0326577)

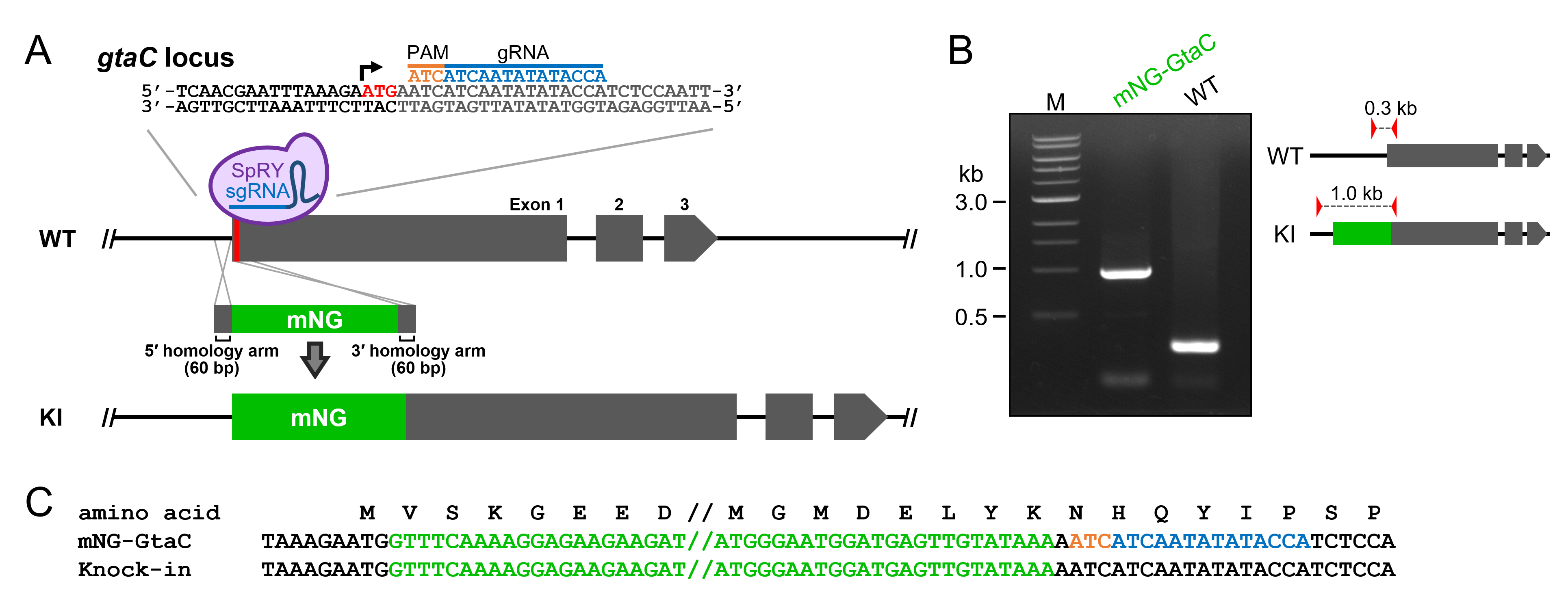

Supplement: S1 Fig — (A) Schematic representation of the knock-in strategy for integrating mNG at the gtaC locus using the CRISPR/Cas9 system. (B) PCR validation of the knock-in strain using primers targeting the regions upstream and downstream of the mNG insertion site. The knock-in strain produced a PCR product with an increased length corresponding to the inserted mNG sequence. (C) Nucleotide sequencing of the gtaC locus in knock-in mutants confirmed successful integration, with the sequence encoding mNG highlighted in green. (TIF) [file pone.0326577.s001.tif]

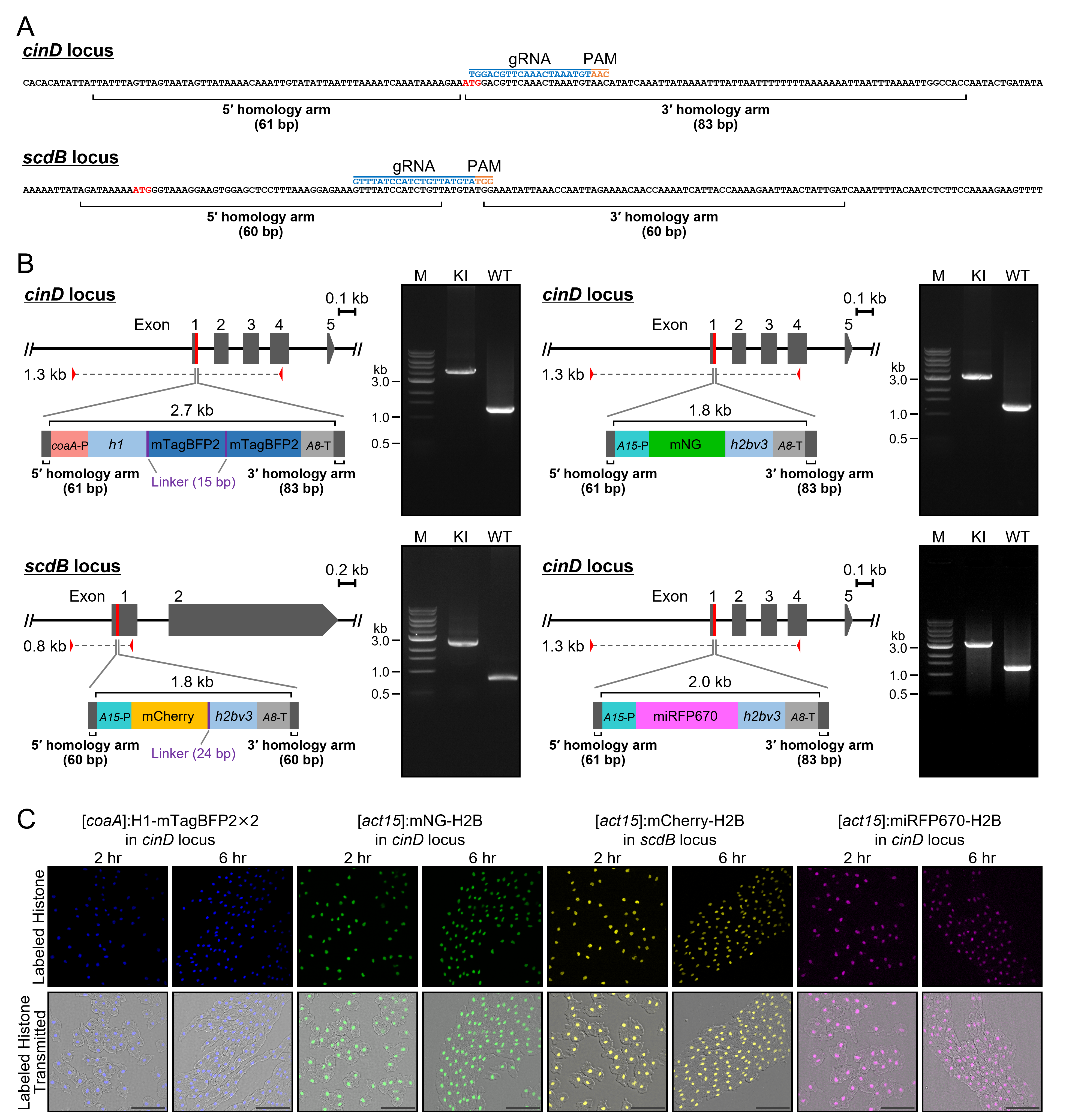

Supplement: S2 Fig — (A) gRNA design for the CRISPR/Cas9 system targeting genomically safe harbour loci. The genomic sequences of cinD and scdB are shown, with the start codon highlighted in red, gRNA sequence in blue, and PAM sequence in orange. Homology arms for the knock-in donor design near the target sites are also displayed. (B) PCR validation of the knock-in strain using primers targeting the regions upstream and downstream of the insertion site. The knock-in strain produced a PCR product with an increased length, corresponding to the size of the inserted knock-in cassette. This knock-in cassette included a histone gene (h1 or h2bv3) fused to one of four fluorescent tags. The expression of these fusion genes is regulated by either the coaA or act15 promoter and is terminated by the act8 terminator. (C) Representative images of nuclear labelling during early development (2 h) and stream formation (6 h). Targeted integration at the safe harbour locus resulted in stable and uniform nuclear labelling without inducing developmental abnormalities. Scale bars: 40 µm. (TIF) [file pone.0326577.s002.tif]

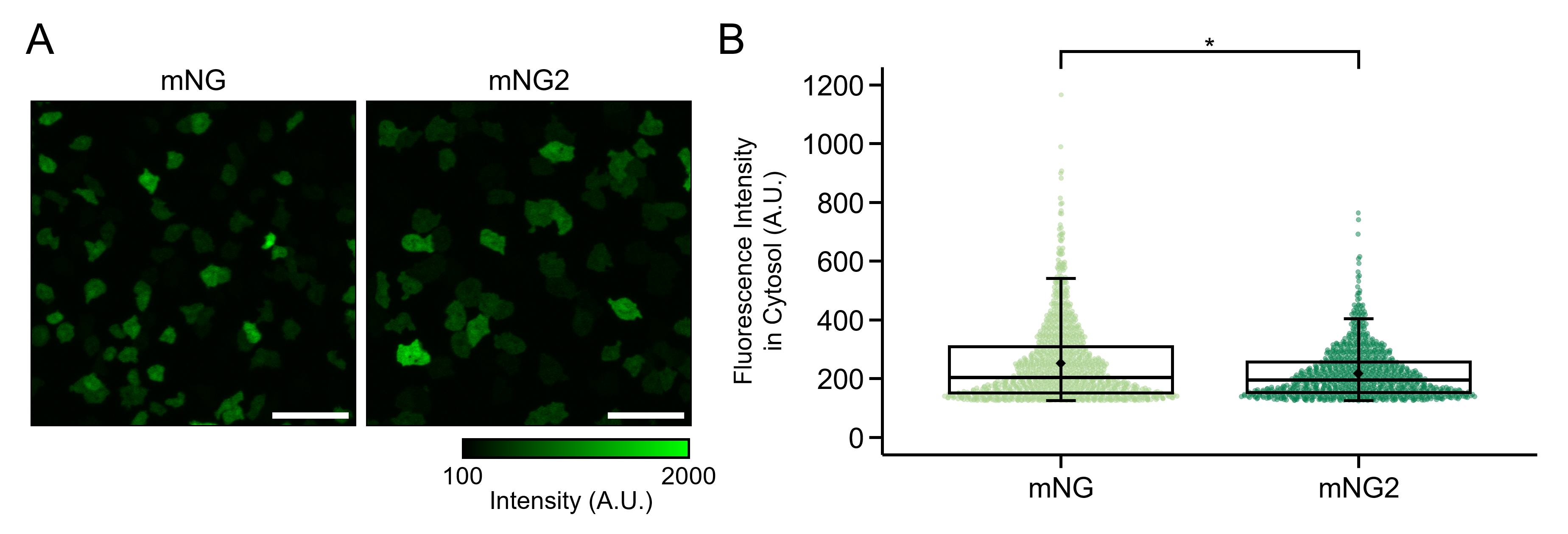

Supplement: S3 Fig — (A) Snapshots of cells overexpressing full-length mNG and mNG2 from plasmids. The images show only the mNG or mNG2. Fluorescence intensity of mNG is indicated by the colour key. Scale bars: 50 µm. (B) Quantification of fluorescence intensity of overexpressed full-length mNG and mNG2. n = 1117 for mNG; 978 for mNG2. *p < 0.001; The Wilcoxon rank-sum test. (TIF) [file pone.0326577.s003.tif]

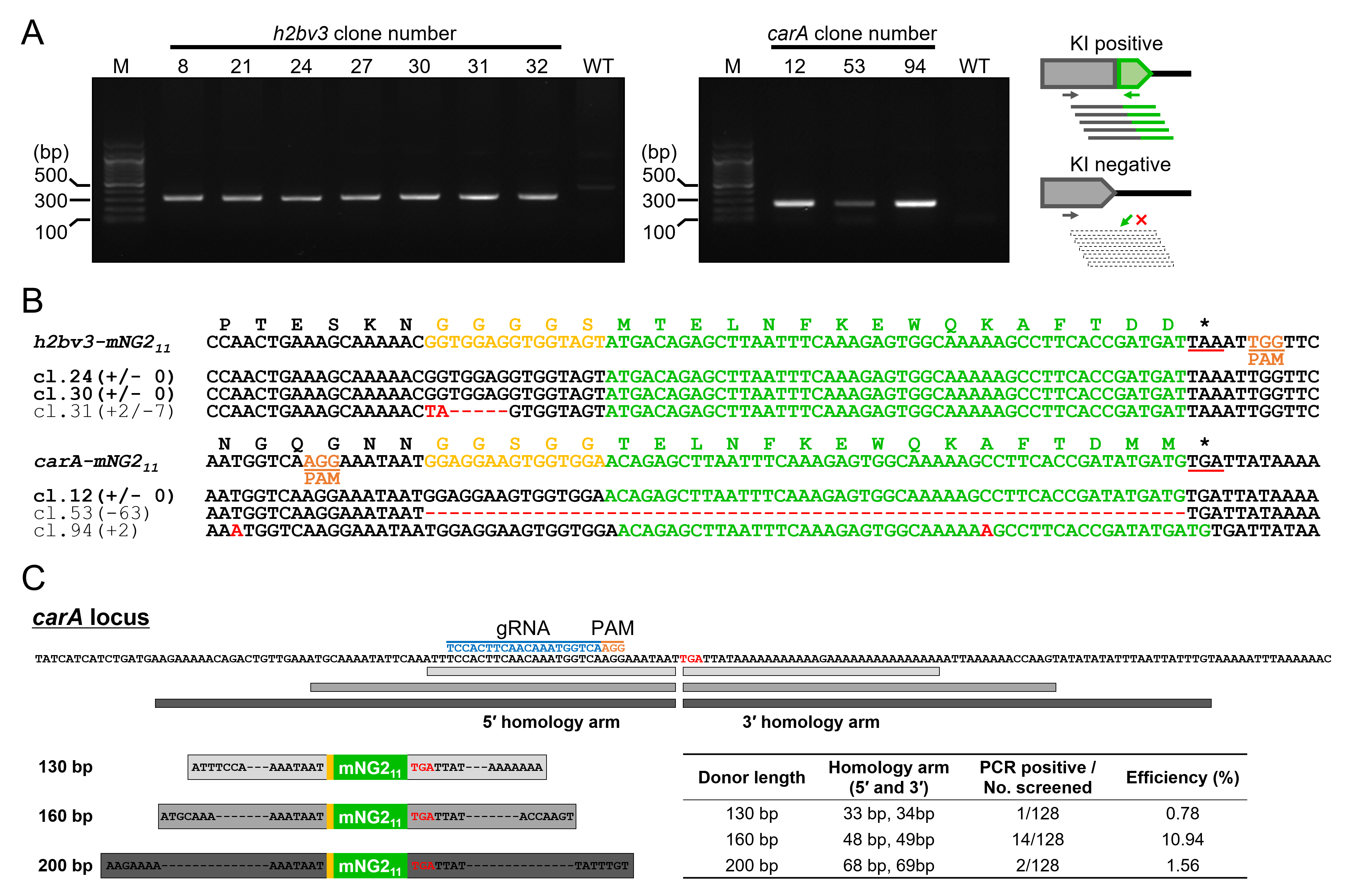

Supplement: S4 Fig — (A) Detection of knock-in events using PCR amplification. Forward primers were designed for the C-terminal region of the target gene and reverse primers within mNG211 were used. Knock-in positive clones were identified based on the presence of PCR-amplified products. (B) Sequence analysis of PCR-positive clones. The stop codon is underlined in red. Linker sequences are highlighted in yellow, mNG211 green; PAM sequences orange; and mutations red. Correct clones are marked in bold, with the number of base errors indicated in parentheses next to the clone number. (C) Comparison of knock-in efficiency based on differences in homologous arm lengths. Donor DNAs were prepared as PCR-amplified double-stranded DNA (dsDNA) fragments with total lengths of 130, 160, or 200 nucleotides. (TIF) [file pone.0326577.s004.tif]

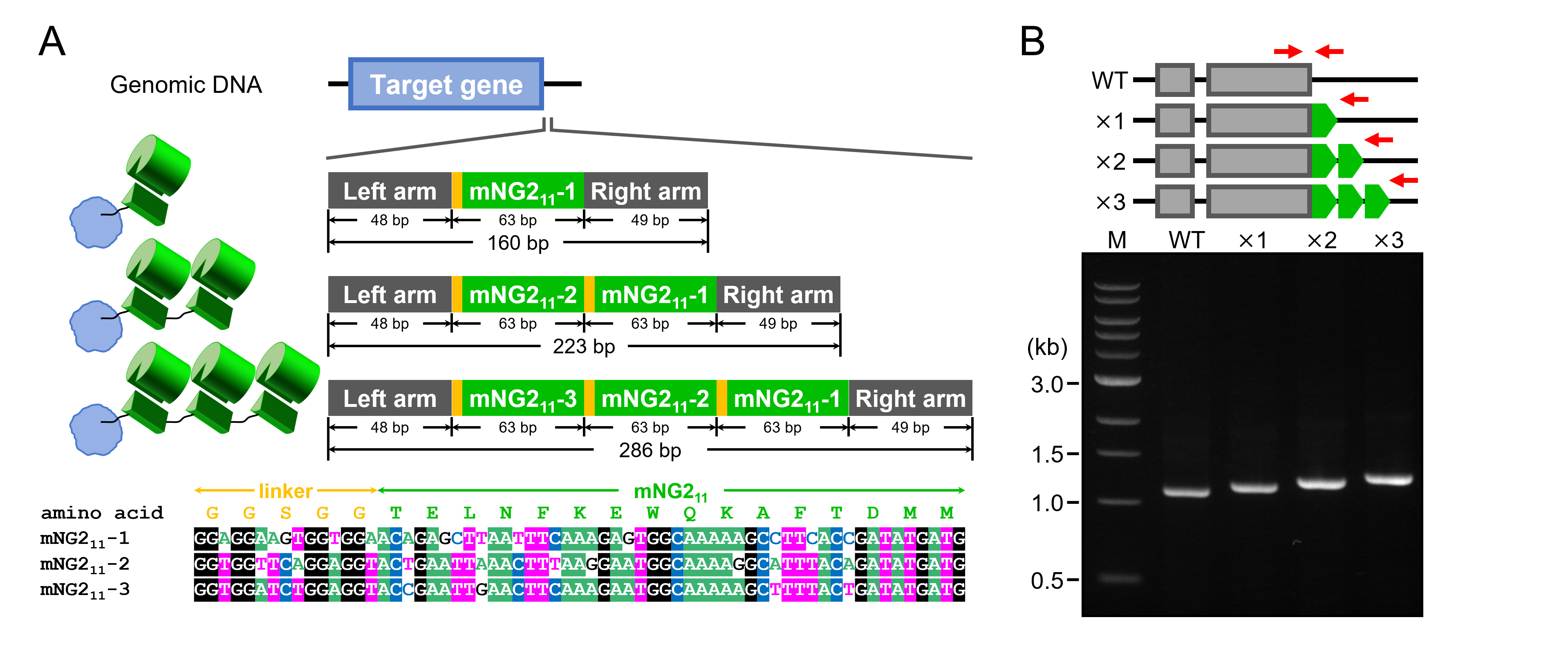

Supplement: S5 Fig — (A) Schematic representation of CRISPR/Cas9-mediated insertion of split mNG211 at the C-terminus of carA. Donor DNAs with varying repeat numbers are shown. (B) PCR-based detection of knock-in events using primers targeting carA and a flanking region outside the gene (red arrows). Expected PCR product sizes: 1,127 bp for WT, 1,190 bp for one repeat, 1,253 bp for two repeats, and 1,316 bp for three repeats. (TIF) [file pone.0326577.s005.tif]

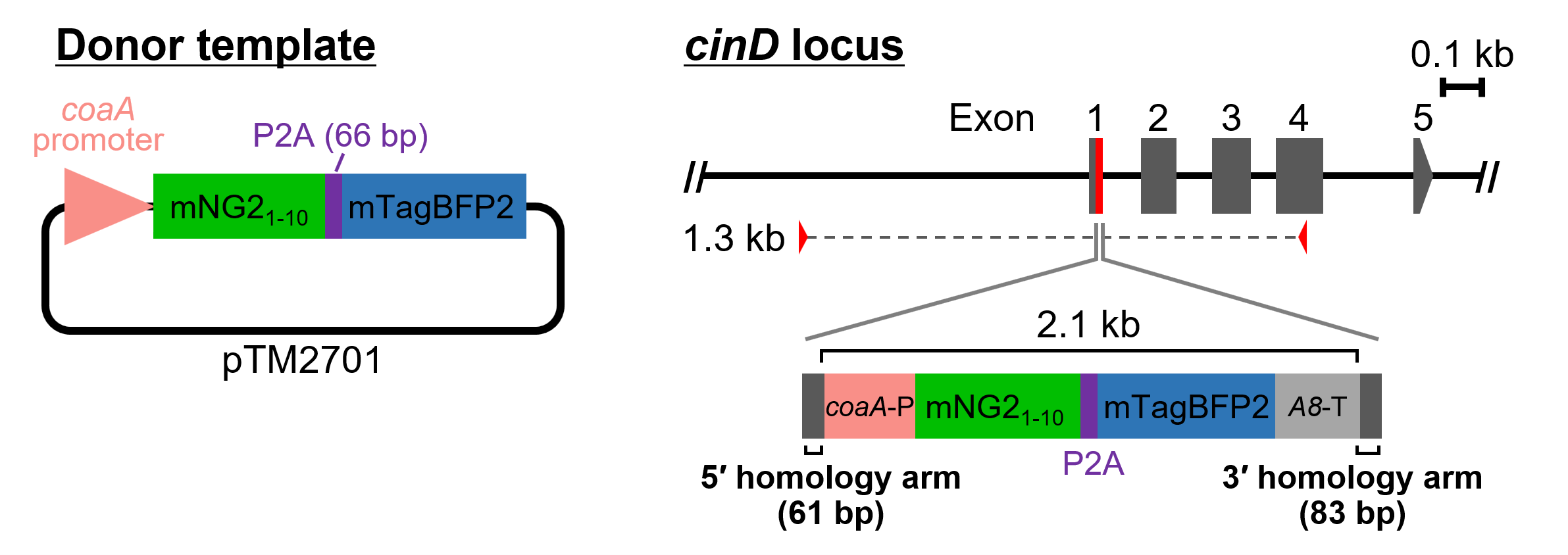

Supplement: S6 Fig — The plasmid vector pTM2701 was constructed to express mNG21–10 under the control of the coaA promoter. Donor DNA generated using this vector as a template was integrated into the cinD locus, enabling stable genomic expression. (TIF) [file pone.0326577.s006.tif]
