# Supplementary material for: Efficient endogenous protein labelling in Dictyostelium using CRISPR/Cas9 knock-in and split fluorescent proteins
Source: PLoS One. 2025 Jun 20;20(6):e0326577. doi: 10.1371/journal.pone.0326577 (PMC12180633; doi:10.1371/journal.pone.0326577)
Supplement: S1 Table — (PDF) [file pone.0326577.s007.pdf]

S1 Table. Codon-optimised nucleotide sequences for mNeonGreen2 and mTagBFP2.

Description	Sequence (5'-3')
**mNeonGreen2**	ATGGTATCAAAAGGAGAGGAGGATAATATGGCTAGTTTACCTGCAACAC<br>ATGAGTTACACATATTTGGATCTATCAATGGTGTTGATTTTGATATGGTA<br>GGACAAGGTACTGGTAATCCTAATGATGGATATGAGGAGCTTAATTTGA<br>AGTCAACTAAGGGAGATTTACAATTTTCTCCTTGGATCTTAGTTCCACA<br>CATCGGATATGGTTTCCATCAATATTTGCCATATCCTGATGGAATGAGTC<br>CATTTCAAGCAGCAATGGTAGATGGATCAGGTTACCAAGTTCACCGTA<br>CTATGCAATTCGAGGATGGTGCTAGTTTGACAGTAAATTACAGATACAC<br>CTACGAAGGATCTCATATCAAGGGTGAGGCACAAGTTATGGGAACAGG<br>TTTCCCTGCCGATGGTCCAGTAATGACTAATACTCTTACCGCCGCAGAT<br>TGGTGTATGTCAAAAAAGACTTATCCTAATGATAAAACAATAATCAGTAC<br>ATTCAAATGGTCTTACACCACCGTTAATGGAAAGAGATATAGATCAACA<br>GCTCGTACCACTTACACATTCGCTAAACCAATGGCAGCTAATTATCTTA<br>AAAATCAACCTATGTATGTATTCAGAAAGACTGAATTAAAACACAGTATG<br>**ACAGAGCTTAATTTCAAAGAGTGGCAAAAAGCCTTCACCGATATGAT**<br>**G**
**mTagBFP2**	ATGGTTAGTAAAGGTGAGGAATTGATTAAGGAGAATATGCACATGAAAC<br>TTTATATGGAAGGAACCGTTGATAATCATCATTTCAAATGTACTTCAGAG<br>GGTGAGGGAAAACCTTATGAAGGAACCCAAACCATGAGAATTAAGGTT<br>GTAGAAGGAGGACCACTTCCATTCGCTTTTGATATACTTGCAACAAGTT<br>TTTTGTATGGATCAAAGACATTTATTAATCACACCCAAGGAATACCAGAT<br>TTTTTTAAGCAATCATTCCCAGAGGGATTCACATGGGAGCGTGTAACC<br>ACTTACGAGGATGGTGGTGTACTTACAGCTACTCAAGATACATCTTTAC<br>AAGATGGATGTTTAATATATAATGTTAAAATTAGAGGAGTAAATTTCACCT<br>CTAATGGACCAGTAATGCAAAAGAAGACTTTGGGTTGGGAGGCATTTA<br>CAGAAACTTTATACCCTGCCGATGGTGGTCTTGAAGGAAGAAATGATAT<br>GGCATTAAAACTTGTAGGAGGTAGTCATTTAATTGCCAATGCTAAAACA<br>ACATACCGTTCAAAGAAACCAGCTAAAAATTTGAAAATGCCTGGAGTTT<br>ACTACGTTGATTACAGATTGGAGAGAATCAAAGAGGCCAATAATGAAAC<br>TTACGTTGAACAACACGAGGTTGCAGTAGCTAGATATTGTGATTTGCCA<br>TCTAAATTAGGTCATAAGTTAAATTAA

The nucleotide sequences corresponding to $mNG2_{11}$ is shown in bold.
