# Supplementary material for: Efficient endogenous protein labelling in Dictyostelium using CRISPR/Cas9 knock-in and split fluorescent proteins
Source: PLoS One. 2025 Jun 20;20(6):e0326577. doi: 10.1371/journal.pone.0326577 (PMC12180633; doi:10.1371/journal.pone.0326577)
Supplement: S2 Table — mNG: mNeonGreen; mTB2: mTagBFP2; Blast: Blasticidin S; Hyg: Hygromycin. (PDF) [file pone.0326577.s008.pdf]

S2 Table. Expression vectors used in this study.

Vector	Description	Backbone	Drug Resistance	Reference
**pDM304**	extrachromosomal expression vector	–	G418	(Veltman et al, 2009)
**pDM358**	extrachromosomal expression vector	–	Hyg	(Veltman et al, 2009)
**pDM1208**	[*act15*]: mCherry	–	G418	(Paschke et al, 2018)
**pTM1931**	[*act15*]: mCherry-H2B	pDM1208	G418	This study
**pTM2035**	[*act15*]: mNG	pDM326	Blast	(Yamashita et al, 2025)
**pTM2036**	[*act15*]: mNG-H2B	pTM2035	Blast	(Yamashita et al, 2025)
**pTM2045**	[*act15*]: mNG	pDM358	Hyg	This study
**pTM2046**	[*act15*]: mNG	pDM304	G418	This study
**pTM2054**	[*act15*]: mNG2	pDM304	G418	This study
**pTM2055**	[*act15*]: mNG2$_{1\text{-}10}$	pDM304	G418	This study
**pTM2066**	[*act15*]: mNG-GtaC	pTM2045	G418	This study
**pTM2119**	[*act15*]: linker-mTagBFP2	pDM326	Blast	This study
**pTM2554**	extrachromosomal expression vector with *coaA* promoter	pDM358	Hyg	(Yamashita et al, 2025)
**pTM2580**	[*act15*]: H1-mTB2	pTM2119	Blast	This study
**pTM2585**	[*act15*]: H1-mTB2×2	pTM2580	Blast	This study
**pTM2595**	[*coaA*]: H1-mTB2×2	pTM2554	Hyg	This study
**pTM2609**	[*act15*]: mNG-H2B	pTM2046	G418	This study
**pTM2638**	[*act15*]: linker-mNG	pDM304	G418	This study
**pTM2652**	[*act15*]: mNG2$_{11}$-H2B	pDM358	Hyg	This study
**pTM2653**	[*act15*]: cAR1-mNG2$_{11}$×1	pDM358	Hyg	This study
**pTM2659**	[*act15*]: cAR1-mNG	pTM2638	G418	This study
**pTM2672**	[*act15*]: mNG2$_{1\text{-}10}$	pDM358	Hyg	This study
**pTM2685**	[*coaA*]: P2A-ATG-mTB2	pTM2554	Hyg	This study
**pTM2690**	[*act15*]: cAR1-mNG2$_{11}$×2	pTM2653	Hyg	This study
**pTM2691**	[*act15*]: cAR1-mNG2$_{11}$×3	pTM2690	Hyg	This study
**pTM2696**	[*coaA*]: P2A-mTB2	pTM2685	Hyg	This study
**pTM2701**	[*coaA*]: mNG2$_{1\text{-}10}$-P2A-mTB2	pTM2696	Hyg	This study
**pTM2783**	H2B-mNG2$_{11}$×2 donor	pMD20	-	This study
**pTM2784**	H2B-mNG2$_{11}$×3 donor	pMD20	-	This study

mNG: mNeonGreen; mTB2: mTagBFP2; Blast: Blasticidin S; Hyg: Hygromycin B
