# Supplementary material for: Efficient endogenous protein labelling in Dictyostelium using CRISPR/Cas9 knock-in and split fluorescent proteins
Source: PLoS One. 2025 Jun 20;20(6):e0326577. doi: 10.1371/journal.pone.0326577 (PMC12180633; doi:10.1371/journal.pone.0326577)
Supplement: S3 Table — Lowercase letters are any three nucleotides or restriction enzyme recognition sites. mNG: mNeonGreen; mTB2: mTagBFP2. (PDF) [file pone.0326577.s009.pdf]

S3 Table. Oligonucleotides for cloning primers.

Primer	Gene	Sequence (5'-3')	Purpose
**P2550**	*mNG*	actggatccATGGTTTCAAAAGGAGAAGAAGATA	Cloning at N-terminus
**P2551**	*mNG*	actagatctTTTATACAACTCATCCATTCCCAT	Cloning at N-terminus
**P3119**	*mNG*	actactagtGGTGGAGGTGGTAGTATGGTTTCAAAAGGAGAAGAA	Cloning at C-terminus with G4S linker
**P2555**	*mNG*	acttctagaTTTATACAACTCATCCATTCCCAT	Cloning at C-terminus with G4S linker
**P2680**	*mTB2*	actactagtGGTGGATCCGGAGGTATGGTTAGTAAAGGTGAGGAAT	Cloning at C-terminus with G4S linker
**P2681**	*mTB2*	acttctagaTTAATTTAACTTATGACCTAATTTAGATGG	Cloning at C-terminus with G4S linker
**P2720**	*mTB2*	acttctagaATTTAACTTATGACCTAATTTAGATGG	Cloning at C-terminus with G4S linker
**P3162**	*mTB2*	actactagtGGATCAGGAGCAACCAATTTCAGTTTGCTTAAACAAGCAGGTGATGTTGAGGAGAATCCAGGTCCTATGGTTAGTAAAGGTGAGGAAT	Cloning at C-terminus with P2A
**P2562**	*mNG2*$_{1\text{-}10}$	actagatctATGGTATCAAAAGGAGAGGAGGATA	Cloning
**P2563**	*mNG2*$_{1\text{-}10}$	agtactagtTTACATACTGTGTTTTAATTCAGTCTTTC	Cloning
**P3203**	*mNG2*$_{1\text{-}10}$	GGATCGTTTCGCAGATCTATGGTATCAAAAGGAGAGGA	Cloning
**P3204**	*mNG2*$_{1\text{-}10}$	GGTTGCTCCTGATCCCATACTGTGTTTTAATTCAGTCTTTC	Cloning
**P601**	*gtaC*	ggatccAAAATAATGAATCATCAATATATACCATCTCCAAT	Cloning
**P611**	*gtaC*	actagtATCGCTAATTAATTTTGAAACACTCATTGCAC	Cloning
**P3039**	*h1*	actagatctATGGGTCCAAAAGCACC	Cloning at C-terminus
**P3040**	*h1*	actactagtTTTTTTGGCAGCGACTT	Cloning at C-terminus
**P2260**	*h2bv3*	actagatctATGGTATTCGTTAAAGGTCAAAAG	Cloning at C-terminus
**P2140**	*h2bv3*	actactagtTTAGTTTTTGCTTTCAGTTGG	Cloning at C-terminus
**P3133**	*h2b-mNG2*$_{11}$	actagatctATGACAGAGCTTAATTTCAAAGAGTGGCAAAAAGCCTTCACCGATATGATGGGAGGAAGTGGTGGAGTATTCGTTAAAGGTCAAAAG	Cloning
**P3117**	*carA*	actagatctATGGGTCTTTTAGATGGAAATC	Cloning at C-terminus
**P3118**	*carA*	actactagtATTATTTCCTTGACCATTTGTTG	Cloning at C-terminus
**P3134**	*carA-mNG2*$_{11}$	actactagtTCACATCATATCGGTGAAGGCTTTTTGCCACTCTTTGAAATTAAGCTCTGTTCCACCACTTCCTCCATTATTTCCTTGACCATTTGTTG	Cloning
**P3179**	*carA*	ATTATTTCCTTGACCATTTGTTG	Inverse PCR

**P3180**	*mNG2$_{11}$* 1st	GGAGGAAGTGGTGGAACA	Inverse PCR
**P3181**	*mNG2$_{11}$* 2nd	GGTCAAGGAAATAATGGTGGTTCAGGAGGTACTGAATTAAACTTTAAGGAATGGCAAAA	Cloning
**P3182**	*mNG2$_{11}$* 2nd	TCCACCACTTCCTCCCATCATATCTGTAAATGCCTTTTGCCATTCCTTAAAGTTTAATTC	Cloning
**P3183**	*mNG2$_{11}$* 2nd	GGTGGTTCAGGAGGTACT	Inverse PCR
**P3184**	*mNG2$_{11}$* 3rd	GGTCAAGGAAATAATGGTGGATCTGGAGGTACCGAATTGAACTTCAAAGAATGGCAAAA	Cloning
**P3185**	*mNG2$_{11}$* 3rd	ACCTCCTGAACCACCCATCATATCAGTAAAAGCTTTTTGCCATTCTTTGAAGTTCAATTC	Cloning
**P3199**	*P2A-mTB2*	CTCACCTTTACTAACAGGACCTGGATTCTCCTC	Inverse PCR
**P3200**	*P2A-mTB2*	GTTAGTAAAGGTGAGGAATTGA	Inverse PCR
**P3332**	*h2bv3-mNG2$_{11}$*×2 left	catgccatcttacaaggtatgactgctgtcaacaagtacaatccaactgaaagcaaaaacGGTGGTTCAGGAGGTACTGAAT	TA cloning
**P3333**	*h2bv3-mNG2$_{11}$*×3 left	catgccatcttacaaggtatgactgctgtcaacaagtacaatccaactgaaagcaaaaacGGTGGATCTGGAGGTACC	TA cloning
**P3334**	*h2bv3-mNG2$_{11}$*×3 right	aaaaaagaaaattggaaactatattttttaaggaatatagttcatttggaaccaatttaCATCATATCGGTGAAGGC	TA cloning

Lowercase letters are any three nucleotides or restriction enzyme recognition sites.
mNG: mNeonGreen; mTB2: mTagBFP2
