# Supplementary material for: Efficient endogenous protein labelling in Dictyostelium using CRISPR/Cas9 knock-in and split fluorescent proteins
Source: PLoS One. 2025 Jun 20;20(6):e0326577. doi: 10.1371/journal.pone.0326577 (PMC12180633; doi:10.1371/journal.pone.0326577)
Supplement: S4 Table — Lowercase letters of the gRNA represent overhang sequences for Golden Gate Assembly. mNG: mNeonGreen. (PDF) [file pone.0326577.s010.pdf]

S4 Table. Oligonucleotides for gRNAs and sequencing primers.

Primer	Gene	Sequence (5'-3')	Purpose
**P2143**	*gtaC*	agcaTGGAGATGGTATATATTGAT	gRNA sense
**P2144**	*gtaC*	aaacATCAATATATACCATCTCCA	gRNA antisense
**P2151**	*gtaC*	AAAATCAATAACCAACTCTCGCA	Screening/sequencing-Fw
**P2152**	*gtaC*	TGGTCGCTATAAATTGGAGATGGT	Screening/sequencing-Rv
**P2263**	*cinD*	agcaTGGACGTTCAAACTAAATGT	gRNA sense
**P2264**	*cinD*	aaacACATTTAGTTTGAACGTCCA	gRNA antisense
**P1186**	*cinD*	GAATCAGTTGTATCTAAAGATGG	Screening/sequencing-Fw
**P155**	*cinD*	AACAGCTTGACTTGGGATGG	Screening/sequencing-Rv
**P2684**	*scdB*	agcaGTTTATCCATCTGTTATGTA	gRNA sense
**P2685**	*scdB*	aaacTACATAACAGATGGATAAAC	gRNA antisense
**P106**	*scdB*	ATTCAAAATGGCATGCAGGT	Screening/sequencing-Fw
**P2648**	*scdB*	GGGGTTAAGTAAGATGGCATGAG	Screening/sequencing-Rv
**P2608**	*carA*	agcaTCCACTTCAACAAATGGTCA	gRNA sense/screening-Fw
**P2609**	*carA*	aaacTGACCATTTGTTGAAGTGGA	gRNA antisense
**P2634**	*carA*	TGATGTGCAATGTAGTTCCGA	Screening/sequencing-Fw/ Sanger sequencing
**P2635**	*carA*	TCTTGTGATGTTTGGCTGTCC	Screening/sequencing-Fw
**P3152**	*carA*	TGAAATTAAGCTCTGTTCCACC	Screening-Rv
**P3083**	*h2bv3*	agcaACTGAAAGCAAAAACTAAAT	gRNA sense
**P3084**	*h2bv3*	aaacATTTAGTTTTTGCTTTCAGT	gRNA antisense
**P3085**	*h2bv3*	ATTACTCCACCAACCAATGC	Screening/sequencing-Fw/ Sanger sequencing
**P3087**	*h2bv3*	ATCCTCTACACCACCTCCCT	Screening/sequencing-Fw
**P3109**	*h2bv3*	TTAAGCTCTGTCATACTACC	Screening-Rv
**P2282**	*mCherry*	TGGGTTGGGAAGCATCATCA	Sanger sequencing
**P1441**	*mCherry*	AGAGGATCCTTTATATAATTCATCC ATAC	Sanger sequencing
**P2168**	*mNG*	GCAGCAATGGTAGATGGTTCAG	Sanger sequencing
**P2167**	*mNG*	ACTTCTACACCAATCAGCAGCTG	Sanger sequencing
**P2260**	*h2bv3*	ACTAGATCTATGGTATTCGTTAAAG GTCAAAAG	Sanger sequencing
**P2140**	*h2bv3*	ACTACTAGTTTAGTTTTTGCTTTCAG TTGG	Sanger sequencing

Lowercase letters of the gRNA represent overhang sequences for Golden Gate Assembly.
mNG: mNeonGreen
