# Supplementary material for: Efficient endogenous protein labelling in Dictyostelium using CRISPR/Cas9 knock-in and split fluorescent proteins
Source: PLoS One. 2025 Jun 20;20(6):e0326577. doi: 10.1371/journal.pone.0326577 (PMC12180633; doi:10.1371/journal.pone.0326577)
Supplement: S5 Table — (PDF) [file pone.0326577.s011.pdf]

S5 Table. CRISPR/Cas9 vectors for generating knock-in mutants.

Plasmid	Target gene	Backbone	Cas9 Type	Reference
**pTM1901**	*gtaC*	pTM1668	SpRY	(Yamashita et al, 2025)
**pTM1933**	*cinD*	pTM1668	SpRY	(Yamashita et al, 2025)
**pTM2123**	*scdB*	pTM1599	SpCas9	This study
**pTM2080**	*carA*	pTM1599	SpCas9	This study
**pTM2600**	*h2bv3*	pTM1285	SpCas9	This study
