# Supplementary material for: Efficient endogenous protein labelling in Dictyostelium using CRISPR/Cas9 knock-in and split fluorescent proteins
Source: PLoS One. 2025 Jun 20;20(6):e0326577. doi: 10.1371/journal.pone.0326577 (PMC12180633; doi:10.1371/journal.pone.0326577)
Supplement: S6 Table — Lowercase letters represent homologous sequences of target genes. mNG: mNeonGreen. (PDF) [file pone.0326577.s012.pdf]

S6 Table. Primers for generating donor DNAs.

Primer	Description	Sequence (5'-3')
**P2261**	*cinD act15*-P left-arm	ttatttagttagtaatagttataaaacaaattgtatattaatttaaaatcaaataaaagaaCATTCTCGAGACTAGAGCTAG
**P2262**	*cinD act8*-T right-arm	ggtggccaattttaaattaatttttttaaaaaaaattaataaattttataatttgatatgttacatttagtttgaacgtccatCTTTTTCGAAATCGATAAGC
**P3030**	*cinD coaA*-P left-arm	ttatttagttagtaatagttataaaacaaattgtatattaatttaaaatcaaataaaagaaCTCGAGAATGATTAATCATATGAC
**P2686**	*scdB act15*-P left-arm	agataaaaaatgggtaaaggaagtggagctcctttaaaggagaaagtttatccatctgttCATTCTCGAGACTAGAGCTAG
**P2687**	*scdB act8*-T right-arm	atcaatagttaattcttttggtaatgattttggttgttttctaattggtttaatatttccCTTTTTCGAAATCGATAAGC
**P2149**	mNG-GtaC left-arm	aacaaccaatcaaataaaacaacaataaggaggagattgtattctcaacgaatttaaagaATGGTTTCAAAAGGAGAAGAAGATA
**P2150**	mNG-GtaC right-arm	tacattgtgtacaccagagttttggtcgctataaattggagatggtatatattgatgattTTTATACAACTCATCCATTCCCAT
**P2659**	cAR1-mNG left-arm	cagactgttgaaatgcaaaatattcaaatttccacttcaacaaatggtcaaggaaataatGGTGGATCCGGAGGTATGGTTTCAAAAGGTGAAGAA
**P3173**	cAR1-mNG right-arm	taaatatatatacttggttttttaattttttttttttttttcttttttttttttataatcaTTTATACAACTCATCCATTCCC
**P3253**	H2B-mNG left-arm	catgccatcttacaaggtatgactgctgtcaacaagtacaatccaactgaaagcaaaaacGGTGGAGGTGGTAGTATGGTTTCAAAAGGAGAAGAAGATA
**P3254**	H2B-mNG right-arm	aaaaaagaaaattggaaactatattttttaaggaatatagttcatttggaaccaatttaTTTATACAACTCATCCATTCCCAT
**P3107**	H2B-mNG2$_{11}$ ssODN	gtcaacaagtacaatccaactgaaagcaaaaacGGTGGAGGTGGTAGTATGACAGAGCTTAATTTCAAAGAGTGGCAAAAAGCCTTCACCGATGATTAAattggttccaaatgaactatattccttaaaa
**P3151**	cAR1-mNG2$_{11}$ ssODN	atttccacttcaacaaatggtcaaggaaataatGGAGGAAGTGGTGGAACAGAGCTTAATTTCAAAGAGTGGCAAAAAGCCTTCACCGATATGATGtgattataaaaaaaaaaagaaaaaaaaaaaaaaa
**P3186**	cAR1-mNG2$_{11}$ left-arm 33 bp	atttccacttcaacaaatggtca
**P3187**	cAR1-mNG2$_{11}$ right-arm 34 bp	ttttttttttttttttcttttttttttttataatcaCATCATATCGGTGAAGGC
**P3188**	cAR1-mNG2$_{11}$ left-arm 48 bp	atgcaaaatattcaaatttccacttc
**P3189**	cAR1-mNG2$_{11}$ right-arm 49 bp	acttggttttttaattttttttttttttttttcttttttttttttataatcaCATCATATCGGTGAAGGC
**P3190**	cAR1-mNG2$_{11}$ left-arm 68 bp	aagaaaaacagactgttgaaatgc
**P3191**	cAR1-mNG2$_{11}$ right-arm 69 bp	acaaataattaaatatatatacttggttttttaattttttttttttttttttcttttttttttttataatcaCATCATATCGGTGAAGGC
**P3335**	H2B-mNG2$_{11}$ left-arm 33 bp	gtcaacaagtacaatccaactg
**P3336**	H2B-mNG2$_{11}$ right-arm 34 bp	ttttaaggaatatagttcatttggaac

Lowercase letters represent homologous sequences of target genes. mNG: mNeonGreen
