# Supplementary material for: Efficient endogenous protein labelling in Dictyostelium using CRISPR/Cas9 knock-in and split fluorescent proteins
Source: PLoS One. 2025 Jun 20;20(6):e0326577. doi: 10.1371/journal.pone.0326577 (PMC12180633; doi:10.1371/journal.pone.0326577)
Supplement: S7 Table — mNG: mNeonGreen; mTB2: mTagBFP2. (PDF) [file pone.0326577.s013.pdf]

S7 Table. Plasmid DNAs used as PCR templates.

Plasmid	Description	Reference
**pTM2035**	mNG	(Yamashita et al, 2025)
**pTM2595**	[*coaA*]: H1-mTB2×2	This study
**pTM2036**	[*act15*]: mNG-H2B	(Yamashita et al, 2025)
**pTM1931**	[*act15*]: mCherry-H2B	This study
**pTM2115**	[*act15*]: miRFP670-H2B	(Yamashita et al, 2025)
**pTM2653**	[*act15*]: cAR1-mNG2$_{11}$×1	This study
**pTM2690**	[*act15*]: cAR1-mNG2$_{11}$×2	This study
**pTM2691**	[*act15*]: cAR1-mNG2$_{11}$×3	This study
**pTM2783**	[*act15*]: H2B-mNG2$_{11}$×2	This study
**pTM2784**	[*act15*]: H2B-mNG2$_{11}$×3	This study
**pTM2701**	[*coaA*]: mNG2$_{1\text{-}10}$-P2A-mTB2	This study

mNG: mNeonGreen; mTB2: mTagBFP2
