# Supplementary material for: Efficient endogenous protein labelling in Dictyostelium using CRISPR/Cas9 knock-in and split fluorescent proteins
Source: PLoS One. 2025 Jun 20;20(6):e0326577. doi: 10.1371/journal.pone.0326577 (PMC12180633; doi:10.1371/journal.pone.0326577)
Supplement: S8 Table — KI: Knock-in; OE: Overexpression; Hyg: Hygromycin B. mNG: mNeonGreen; mTB2: mTagBFP2. (PDF) [file pone.0326577.s014.pdf]

S8 Table. Cell lines used in this study.

Strain	Description	Parental Strain	Vectors Used	Target Gene	Drug Resistance
**AX3**	*Dictyostelium discoideum* Parental strain	–	–	–	non
**dTM1482**	[*act15*]: mCherry-H2B KI	Ax3	pTM1933	*cinD*	non
**dTM1588**	mNG-GtaC KI	dTM1482	pTM1901	*gtaC*	non
**dTM1697**	[*act15*]: mNG-GtaC OE	dTM1482	pTM2066	–	Hyg
**dTM1765**	[*act15*]: mNG	dTM1482	pTM2045	–	Hyg
**dTM2107**	cAR1-mNG KI	Ax3	pTM2080	*carA*	non
**dTM2283**	H2B-mNG KI	Ax3	pTM2600	*h2bv3*	non
**dTM1711**	[*act15*]: mNG-H2B KI	Ax3	pTM1933	*cinD*	non
**dTM2019**	[*coaA*]: H1-mTB2×2 KI	Ax3	pTM1933	*cinD*	non
**dTM1708**	[*act15*]: mCherry-H2B KI	Ax3	pTM2123	*scdB*	non
**dTM2015**	[*act15*]: miRFP670-H2B KI	Ax3	pTM1933	*cinD*	non
**dTM2123**	[*act15*]: mNG	dTM1482	pTM2046	–	G418
**dTM2124**	[*act15*]: mNG2	dTM1482	pTM2054	–	G418
**dTM1998**	[*act15*]: $mNG2_{1\text{-}10}$	Ax3	pTM2055	–	G418
**dTM2058**	[*act15*]: $mNG2_{11}$-H2B	Ax3	pTM2652	–	Hyg
**dTM2034**	[*act15*]: $mNG2_{11}$-H2B	dTM1998	pTM2652	–	G418/Hyg
**dTM2055**	[*act15*]: mNG-H2B	Ax3	pTM2609	–	G418
**dTM2059**	[*act15*]: cAR1-$mNG2_{11}$	Ax3	pTM2653	–	Hyg
**dTM2035**	[*act15*]: cAR1-$mNG2_{11}$	dTM1998	pTM2653	–	G418/Hyg
**dTM2057**	[*act15*]: cAR1-mNG	Ax3	pTM2659	–	G418
**dTM2011**	H2B-$mNG2_{11}$ KI	Ax3	pTM2600	*h2bv3*	non
**dTM2021**	[*act15*]: $mNG2_{1\text{-}10}$	dTM2011	pTM2055	–	G418
**dTM2341**	H2B-$mNG2_{11}$×2 KI	Ax3	pTM2600	*h2bv3*	non
**dTM2349**	[*act15*]: $mNG2_{1\text{-}10}$	dTM2341	pTM2055	–	G418
**dTM2063**	cAR1-$mNG2_{11}$ KI	Ax3	pTM2080	*carA*	non
**dTM2064**	[*act15*]: $mNG2_{1\text{-}10}$	dTM2063	pTM2672	–	Hyg
**dTM2160**	[*coaA*]: $mNG2_{1\text{-}10}$-P2A-mTB2	dTM2063	pTM2701	–	Hyg
**dTM2163**	cAR1-$mNG2_{11}$×2 KI	Ax3	pTM2080	*carA*	non
**dTM2165**	[*act15*]: $mNG2_{1\text{-}10}$	dTM2163	pTM2672	–	Hyg
**dTM2167**	[*coaA*]: $mNG2_{1\text{-}10}$-P2A-mTB2	dTM2163	pTM2701	–	Hyg
**dTM2180**	cAR1-$mNG2_{11}$×3 KI	Ax3	pTM2080	*carA*	non
**dTM2183**	[*act15*]: $mNG2_{1\text{-}10}$	dTM2180	pTM2672	–	Hyg
**dTM2185**	[*coaA*]: $mNG2_{1\text{-}10}$-P2A-mTB2	dTM2180	pTM2701	–	Hyg
**dTM2208**	[*coaA*]: $mNG2_{1\text{-}10}$-P2A-mTB2 KI	dTM2180	pTM1933	*cinD*	non

mNG: mNeonGreen; mTB2: mTagBFP2; KI: Knock-in; OE: Overexpression; Hyg: Hygromycin B
