# Supplementary material for: Efficient endogenous protein labelling in Dictyostelium using CRISPR/Cas9 knock-in and split fluorescent proteins
Source: PLoS One. 2025 Jun 20;20(6):e0326577. doi: 10.1371/journal.pone.0326577 (PMC12180633; doi:10.1371/journal.pone.0326577)
Supplement: S1 Raw images — (A) The original gel for S1B Fig. (B) The original gel for Fig S2B. (C) The original gel for Fig S4A. (D) The original gel for S5B. (PDF) [file pone.0326577.s015.pdf]

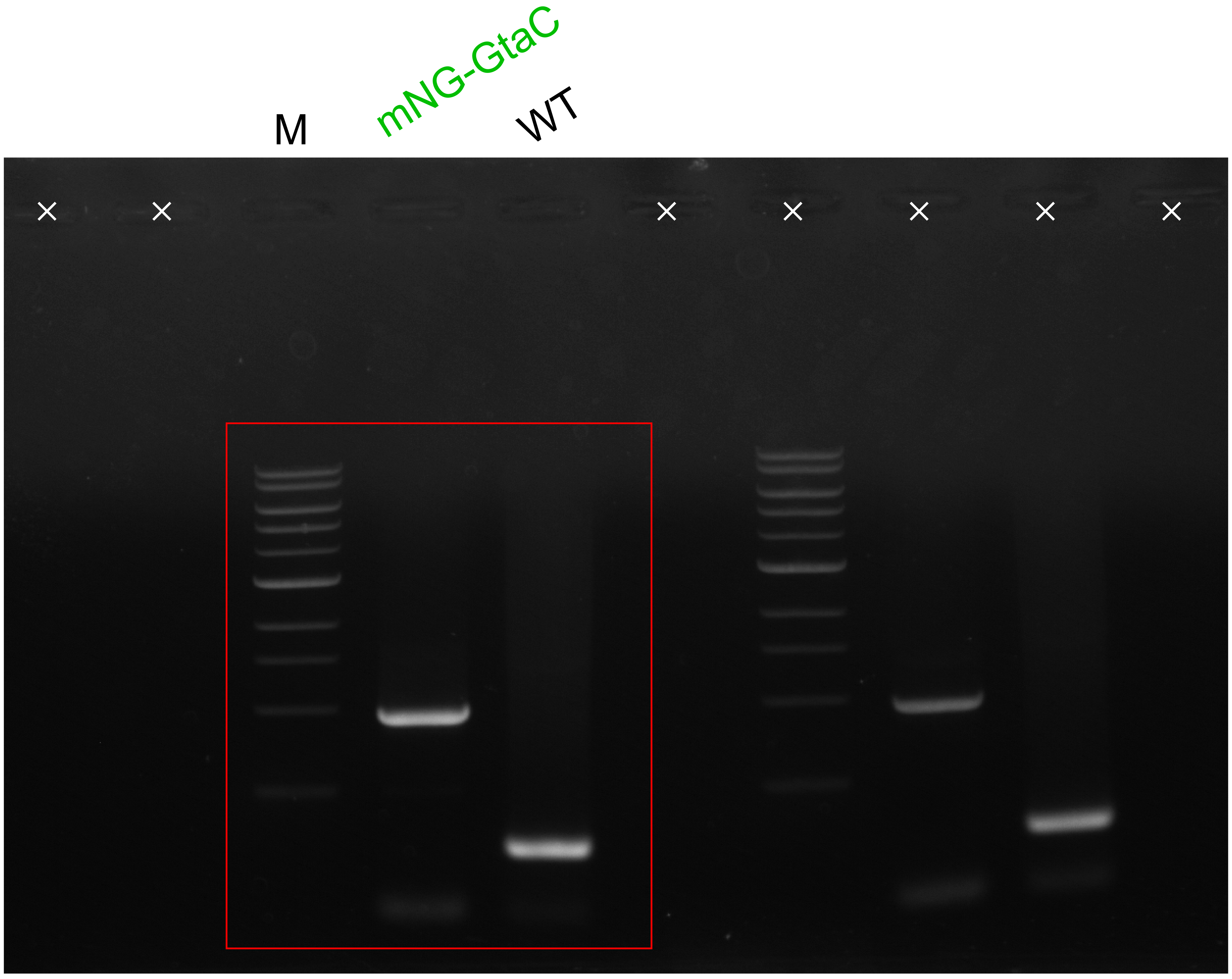

M: 1 kb DNA Ladder (NEB)

(A) The original gel for S1B Fig.

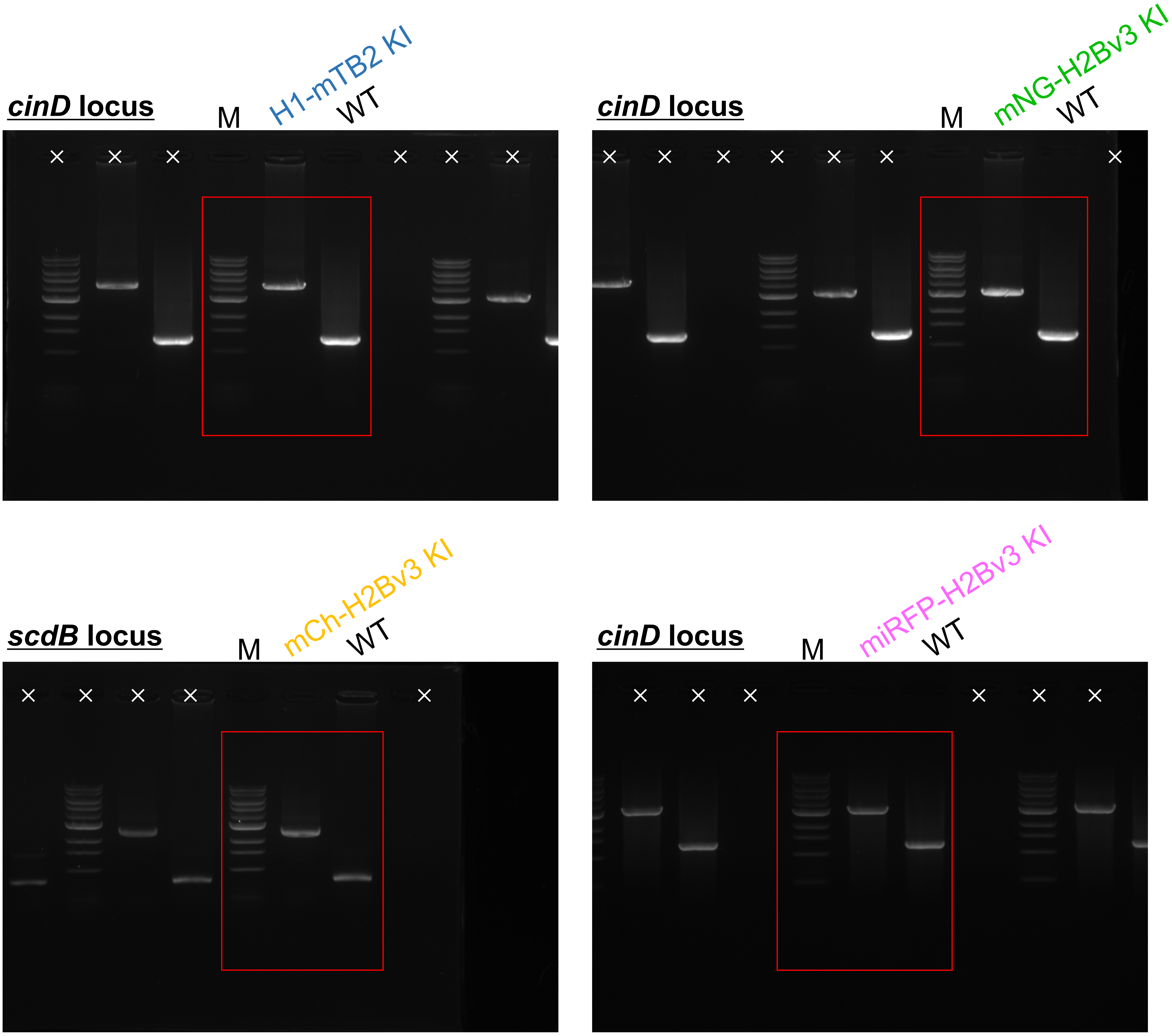

M: 1 kb DNA Ladder (NEB)

(B) The original gel for S2B Fig.

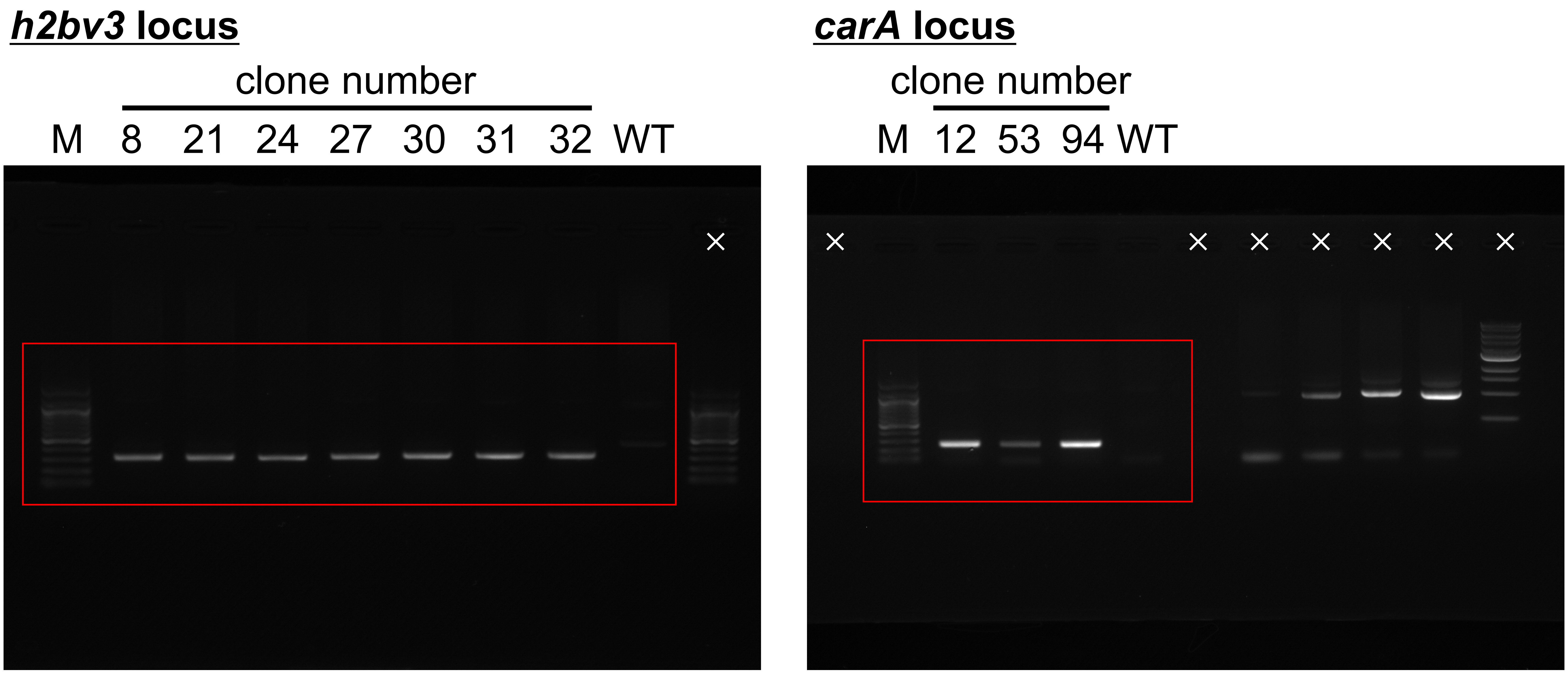

M: 100bp DNA Ladder One (Nacalai)

(C) The original gel for S4A Fig.

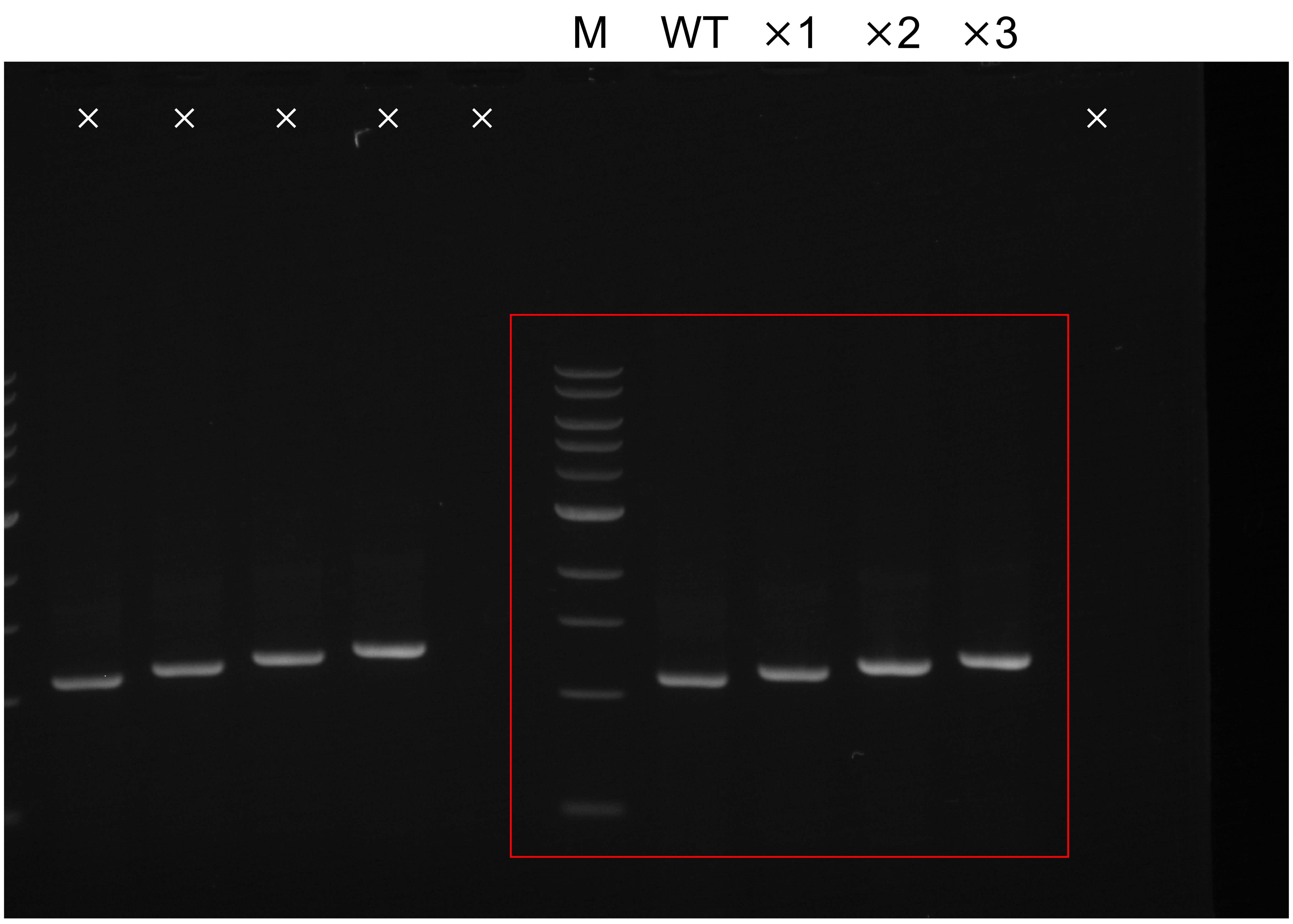

M: 1 kb DNA Ladder (NEB)

(D) The original gel for S5B Fig.
